# Toll-like receptors in fish: evolution, structure, immune signaling, and translational prospects for adjuvant-based vaccine design in aquaculture

**DOI:** 10.3389/fimmu.2026.1880904

**Published:** 2026-07-28

**Authors:** Isabella A. Davis, Kavi R. Miryala, Roy Curtiss, Banikalyan Swain

**Affiliations:** Department of Infectious Diseases and Immunology, College of Veterinary Medicine, University of Florida, Gainesville, FL, United States

**Keywords:** aquaculture vaccines, fish immunology, immune signaling, innate immunity, mucosal immunity, pattern recognition receptors, teleost immunity, toll-like receptors

## Abstract

Toll-like receptors (TLRs) are central to innate immunity and the most studied class of pattern recognition receptors (PRRs). Upon recognition of pathogen-associated molecular patterns (PAMPs) from bacteria, viruses, fungi, and parasites, strong immune responses that bridge innate and adaptive immunity are initiated. Their ability to sense various ligands has made TLR agonists promising candidates for adjuvant development. Unlike traditional adjuvants, TLR ligands directly activate immune pathways, leading to strong, specific, and lasting protection. In aquaculture, there is a critical need for vaccines to reduce reliance on antimicrobials and to address losses from infectious diseases that threaten global food security. We compared TLRs across different teleost and higher vertebrate species for a comprehensive understanding of these receptors. This review focused on defining nomenclature, exploring its historical and evolutionary significance, and structural modeling using computational methods. Using channel catfish (*Ictalurus punctatus*) as a model, we depict two- and three-dimensional structures of various TLRs as well as three-dimensional ligand binding simulations of select catfish TLRs and their associated ligands. These in silico models reveal functional domains and interactions that help predict ligand recognition profiles, binding capacity, and downstream signaling potential. By highlighting the structural features that influence TLR–ligand interactions, our work offers a basis for choosing and improving TLR agonists as next-generation adjuvants in fish vaccines. These findings enhance our understanding of fish immunology and beyond, opening opportunities to develop safer and more effective disease-control techniques in aquaculture through a comparative immunological lens. Together, these findings provide a direct framework for selecting and optimizing TLR agonists, particularly flagellin, CpG ODNs, and poly I:C, as next-generation adjuvants in species-specific aquaculture vaccines.

## 1 Introduction

The immune system is essential for the body’s response to foreign objects, infection, and damage within the host. It encompasses both the innate and acquired immune systems, which are engaged at various stages during an immune response. The innate immune system is defined as a nonspecific first line of defense to infection and is composed of a variety of physical and chemical barriers, cell-mediated processes, and humoral components that often precede and initiate the acquired immune system. Acquired immunity is similarly composed of cell-mediated and humoral components that create a long-lasting “memory” to the infection (1). It has been noted that fish adaptive immune responses are generally less developed than their innate immune responses, as compared to higher vertebrates like mammals. There is evidence that these innate defenses have expanded in the teleost family over evolutionary history, as is the case with the subject of this review, toll-like receptors (TLRs) (2). TLRs are categorized as pattern recognition receptors (PRRs). PRRs are a special class of intracellular or extracellular innate immune receptors that are responsible for responding to a wide variety of pathogen associated molecular patterns (PAMPs) originating from pathogenic infection or damage associated molecular patterns (DAMPs) originating from within the host. Within the PRR family, there are TLRs, nucleotide-binding oligomerization domain (NOD)-like receptors (NLRs), RIG-like receptors (RLRs), and C-type lectin receptors (CLRs) (3). Amongst these PRRs, TLRs are the most well understood and documented across various taxa due to their critical role in bridging the innate and adaptive immune systems by inducing the expression of various proinflammatory cytokines, chemokines, and interferons upon activation by PAMPs (4).

TLRs are type 1 transmembrane proteins localized extracellularly in the outer membranes and intracellularly in the endosomal membranes of immune and non-immune cells; with functional variations in novel fish isoforms (4). TLR signaling is often described as pro-inflammatory, ultimately leading to the release of pro-inflammatory cytokines that further mobilize immune defenses during active infection; however, TLR signaling may also be negatively regulated, which allows for control against over-activation of immune responses that could result in autoimmunity (5). Much of this negative regulation is due to the crosstalk between signaling pathways, such as those mediated by other PRR family receptors (6). TLR structure is fairly conserved across fish and mammals, with three main structural domains: a toll/interleukin-1 receptor (TIR) domain, a transmembrane (TM) region, and a leucine-rich repeat (LRR) domain. The cytoplasmic-facing C-terminus of TLRs contains the TIR domain, where protein-protein interactions occur, allowing for binding of adaptor molecules that initiate downstream signaling cascades. TLRs are anchored to the plasma membrane by their TM regions, which mediate dimerization, a process critical for the activation of certain TLR complexes. TLRs contain a conserved LRR region where ligand binding occurs. The LRR region has a C-terminal end closer to its TM region and an N-terminal extracellular end, creating a unique, horseshoe-shaped solenoidal structured domain composed of alpha-helices and beta-pleated sheets forming a concave surface for binding (4).

Although structural composition tends to be fairly conserved across the families, variations in the number of LRRs and the absence of key functional domains in fish species contribute to their increased diversity and more complex isoform conformations, such as expanded dimer formations and soluble variants. Variations in the LRR count often impact ligand specificity, but deleterious mutations to the LRR sequence result in a loss of function of the whole receptor and the existence of pseudogenes in some fish species (7, 8). Functional TLRs may lack whole TM and TIR domains for negative regulation of inflammation, a feature more common in fish than mammals that will be explored in later sections (9). TLR signaling in fish and mammals functions through two distinct pathways: the myeloid differentiation primary response 88 (MyD88)-dependent pathway and the TIR-domain-containing adapter-inducing interferon-β (TRIF) dependent pathway. Most TLRs signal through the MyD88-dependent pathway, which relies on the binding of adaptor protein MyD88 to initiate pro-inflammatory signaling cascades, inducing cytokines that drive acute inflammatory responses (9, 10). Alternatively, select TLRs signal through the TRIF-dependent pathway that initiates signaling cascades by the association between the TIR domain and adaptor protein TRIF, which is responsible for the production of Type-1 interferons (IFNs), more specific to antiviral and long-term immune responses (11). The culmination of both pathways is the release of transcription factor nuclear factor-kappa light-chain-enhancer of activated B cells (NF-κB), activator protein-1 (AP-1) by mitogen-activated protein kinases (MAPKs) signaling, and interferon regulatory factors (IRFs) by exclusively the TRIF pathway (12, 13). The MAPK family is a class of serine/threonine protein kinases that, like NF-κB, translocate to the nucleus to regulate gene transcription and the expression of pro-inflammatory cytokines involved in the proliferation of immune cells, induce apoptosis, and stress responses in fish necessary for survival (14).

TLRs are organized into 6 phylogenetic families across mammalian and fish species: TLR family 1, 3, 4, 5, 7, and 11. This categorization is based on sequence conservation, ligand specificity, and localization (15). Localization is what drives ligand specificity, with extracellularly located families like TLR families 1, 4, and 5 binding primarily exogenous ligands like lipopeptides and bacterial lipopolysaccharide (LPS); whereas endogenous TLRs in families 3 and 7 recognize internal ligands such as single-stranded RNA (ssRNA) or CpG DNA motifs (16). TLR family 11 has members of both endogenous and membrane-surface localizations. 13 TLRs have been identified and predominantly well characterized in mammals due to their importance for human health applications, whereas there have been more than 21 TLRs discovered across teleost species that are not yet functionally well understood (17). Mammalian homologs exist across fish species, but their expanded number of functional isoforms is due to a number of gene duplications and species variation events that have occurred across evolutionary history. The result is an increase in fish-specific and non-specific TLR paralogs that boost teleost innate immune defenses in response to the constant exposure to pathogens in a free-flowing environment.

TLRs were initially discovered in the invertebrate species of the common fruit fly (*Drosophila melanogaster*) for their developmental role in embryogenesis, but focus was quickly shifted to characterizing their immunological role within mammalian species like mice and humans, as well as observing their broad expression across amphibians, birds, reptiles, other invertebrates, and fish, the subject of this review (18). The specific historical discoveries that have expanded TLR understanding in mammals and fish will be further explored in the history section of this review. We attempt to characterize the role of TLRs as immunological targets by standardizing nomenclature, exploring their historical and evolutionary perspectives, signaling, and structure across the six TLR families. By exploring structural modeling and molecular docking approaches to simulate and optimize adjuvant–TLR interactions, we establish the effectiveness of enabling high-throughput virtual screening to offer reliable structural hypotheses that can be confirmed through experimental validation.

## 2 Nomenclature and discovery timeline

Our knowledge of fish TLRs has been largely shaped through a mammalian discovery lens. Although TLRs remain fairly conserved in their evolutionary history, structural features, and immunological roles, there are a few key differences that are worth discussing.

This section will examine these differences by explaining the systems of nomenclature between mammalian and fish TLRs that currently exist, as well as how inconsistencies between the two have resulted in instances of imprecise naming and classifications of TLRs. We will also discuss the mammalian and fish-specific discoveries that have redefined their role throughout history, beginning with their discovery in 1985 and the findings leading up to our current investigations of their modern role in aquaculture disease management. By better understanding their historical origins and the naming trends between teleost and various phyla, we provide a strong basis for their application in immune signaling and targeting investigations for veterinary medicine and aquaculture applications.

### 2.1 Nomenclature

Early discoveries of mammalian TLRs and subsequent advancements in the classification of human TLRs have shaped the systems of nomenclature and organization of TLRs in fish and other vertebrates. This influence is due to the central role TLRs play in human health, particularly in the pathogenesis and therapeutic targeting of major infectious diseases, cancers, neurodegenerative disorders such as Alzheimer’s disease, and autoimmune diseases like rheumatoid arthritis (19). Nomenclature databases for mammalian species, such as the HUGO Gene Nomenclature Committee (HGNC) website for humans and the Mouse Genome Informatics (MGI) database for mice, have guided the creation of select databases for important aquaculture species (20–23). However, because TLR discoveries in teleost and cartilaginous fish, including elasmobranchs such as sharks and rays, followed those in mammals by several years and progressed at varying rates across fish groups, discrepancies in the naming of certain TLRs have emerged both within and across different fish species.

TLRs were originally sequentially named in the order in which they were discovered and organized into families based on amino acid sequence similarity, functionality, and localization (24). Mutations within the extracellular region affecting LRR number, length, and specific sequence are attributed to these changes in ligand specificity and affinity; therefore, TLRs with similarities within their LRR regions are typically placed within the same families (25). TLRs are divided into two categories across phyla by examining the number of cysteine clusters within their LRR region located at their N and C terminals. Organisms containing only one cysteine cluster at their C-terminal end are more often found in vertebrates and classified as single cysteine cluster TLRs (sccTLRs) or vertebrate-type (V-Type), such as fish; whereas those with multiple cysteine clusters are more often found in invertebrate species and are known as multiple cysteine cluster TLRs (mccTLR) or protostome type (P-type) (26, 29).

TLRs were originally identified in the invertebrate species *D. melanogaster* and quickly explored in humans, where the first Drosophila TLR homolog (dToll) found on human chromosome 9 was designated “hToll” (30). hToll was eventually formally reclassified to TLR4 once functional characterization across species was better understood (27). Within specific literature, TLRs from different species are distinguished by referring to them by abbreviations of their names, such as zfTLR or LrTLR, which represent zebrafish *(Danio rerio)* TLR and rohu (*Labeo rohita*) TLR, respectively (17). As described earlier, mammalian TLRs dominate the literature due to their importance for human health applications, and the full sequencing of their TLRs by 2001 served as a practical reference for the first years of fish TLR discovery (28).

The HUGO Gene Nomenclature Committee (HGNC) website describes the ten human TLRs simply as TLRs 1-10; a standardization that the literature surrounding fish TLRs tends to follow fairly closely, with added 1–2 letter abbreviations indicating divergent isoforms or components of hetero/homodimers (22). Examples of these isoform abbreviations in fish include TLR4a, indicating dimerization with another copy, TLR5M, for membrane localization, TLR5s, for solubility, etc. These examples reflect the instances of alternative splicing, duplication events, and evolutionary divergences that create different expressions of the same gene in fish that are absent from human TLRs (17).

Apart from the HGNC database, there exist limited fish species-specific resource centers, including genetic resources for Japanese rice fish (*Oryzias latipes*) within the National Bioresearch Project (NBRP), and Zebrafish within the Zebrafish Information Network (ZFIN), as well as more general websites like FishDB that exist exclusively for fish genomics (20, 21). ZFIN follows the HGNC’s sequential nomenclature style with TLRs aptly named TLRs 1-5, 7-9, 13-14, 18-23, and 25-28. Within the zebrafish genome, duplicated TLRs and isoforms exist, such as TLR8a and TLR8b, TLR19.1 and TLR19.2, and TLR5a and TLR5b, all of which contribute to receptor dimerization and the diversification of innate immune signaling in teleost species. Other important fish species are often compared with zebrafish due to their established reliance as a research model critical to translational discovery.

One limitation with these knowledge banks is that ZFIN is the only species-specific site that provides a comprehensive, standardized list of all its TLRs, alongside other genes. Furthermore, across broader databases, discrepancies and inconsistencies in the naming of homologous TLRs have been reported between species and even across phyla, reflecting ongoing discoveries in sequence variation and functional characterization. A study by Carlson et al. (29) discussed the discrepancies in nomenclature between vertebrates and revealed several examples, including how teleost TLR14, which had been widely considered to be fish-specific, has since been found in the genus Xenopus, and subsequently renamed to TLR 2 in other public databases (29). The same study also touched on the relationship between inconsistencies in correct TLR classification of lower vertebrate species like fish and their evolutionary distance from their mammalian counterparts. This can be attributed to the fact that literature simply tends to focus on mammals more often in human medicine, and thus, there is less consistency in naming between classes of fish worldwide, which requires higher levels of standardization. For example, TLR14 gets further confused by being renamed to TLR18 in the zebrafish and Japanese pufferfish (*Takifugu rubripes*) species only (21, 29).

Variation in TLR nomenclature among fish is greatly influenced by the substantial diversity in TLR number, classification, and functionality across teleost, often preventing the use of a universally standardized naming system. Standardizing nomenclature is still, however, key to ensuring clear and accurate communication across literature, facilitating cross-species comparisons, and integrating high throughput molecular data seamlessly. For the purposes of this article, channel catfish were selected as the model organism for TLR visualization and simulation due to their extensive characterization in the literature and their importance within the aquaculture industry. Accordingly, we will refer to the catfish TLR isoforms mentioned in this paper using the hyphenated nomenclature (e.g., TLR5–1 rather than TLR5.1), which is the standard for channel catfish as established by Quiniou et al. (2013) (8). Channel catfish TLR nomenclature, amongst other important information, is further organized in **Table 1** for accessible referencing. Mentions of other species of fish TLRs maintain the common gene name as defined by the NCBI database.

**Table 1:** Channel catfish (*Ictalurus punctatus*) TLR summary including family, synonyms, ligands, heterodimerization, and primary signaling pathway [MyD88-Dependent (MyD88) or TRIF-Dependent (TRIF)].

Family	Name	Past/alternate names	Ligand	Hetero-dimerization	Signaling pathway	Sources
1	TLR1	–	Triacylated lipopeptides	TLR1/2	MyD88	(74, 91–93)
1	TLR2	–	LTA	TLR1/2	MyD88	(94–97)
1	TLR18	TLR14	LPS, PGN, and LTA	–	MyD88	(111–113)
1	TLR25	–	unknown	–	MyD88	(116–118)
3	TLR3	–	dsRNA, poly I:C	–	TRIF	(119–123)
4	TLR4-1	TLR4 TLR4.1	High LPS levels, Heat shock proteins (HSPs)	TLR4-1/4-2	MyD88/TRIF	(99, 124–129)
4	TLR4-2	TLR4s TLR4.2	High LPS levels, Heat shock proteins (HSPs)	TLR4-1/4-2	N/A	(99, 124–129)
5	TLR5-1	TLR5.1	Flagellin	TLR5-1/TLR5-2	MyD88	(130, 138)
5	TLR5-2	TLR5.2	Flagellin	TLR5-1/TLR5-2	MyD88	(130, 138)
5	TLR5s	–	Flagellin	–	N/A	(135)
7	TLR7	–	ssRNA, dsRNA, imidazoquinoline	TLR7/8	MyD88	(88, 99, 145–151)
7	TLR8-1	–	ssRNA, imidazoquinoline	TLR7/8, TLR8-1/TLR8-2	MyD88	(88, 99, 145–151)
7	TLR8-2	–	ssRNA, imidazoquinoline	TLR8-1/TLR8-2	MyD88	(88, 99, 145–151)
7	TLR9	–	CpG Motifs	–	MyD88	(152–157)
11	TLR19	–	dsRNA, poly I:C	–	TRIF/MyD88	(7, 161–163)
11	TLR20-1	TLR20	dsRNA, poly I:C	–	TRIF/MyD88	(8, 71, 164–166)
11	TLR20-2	TLR20.1	N/A	–	N/A	(8, 71, 164–166)
11	TLR21	–	CpG Motifs, LPS, poly I:C	–	TRIF?	(167–173)
11	TLR22	–	dsRNA, poly I:C, various bacterial ligands	–	MyD88	(50, 174–176)
11	TLR26	–	Various viral/bacterial ligands-needs better evaluation	–	?	(17, 182)

The “?” symbols in certain boxes within the table indicate that the metric is either unknown or requires further experimentation to determine. The not-applicable (N/A) abbreviation is used to indicate that a receptor is incapable of filling this metric, often due to a structural dimorphism causing a loss of function.

### 2.2 Discovery timeline

#### 2.2.1 Mammalian discovery

**Figure 1:**
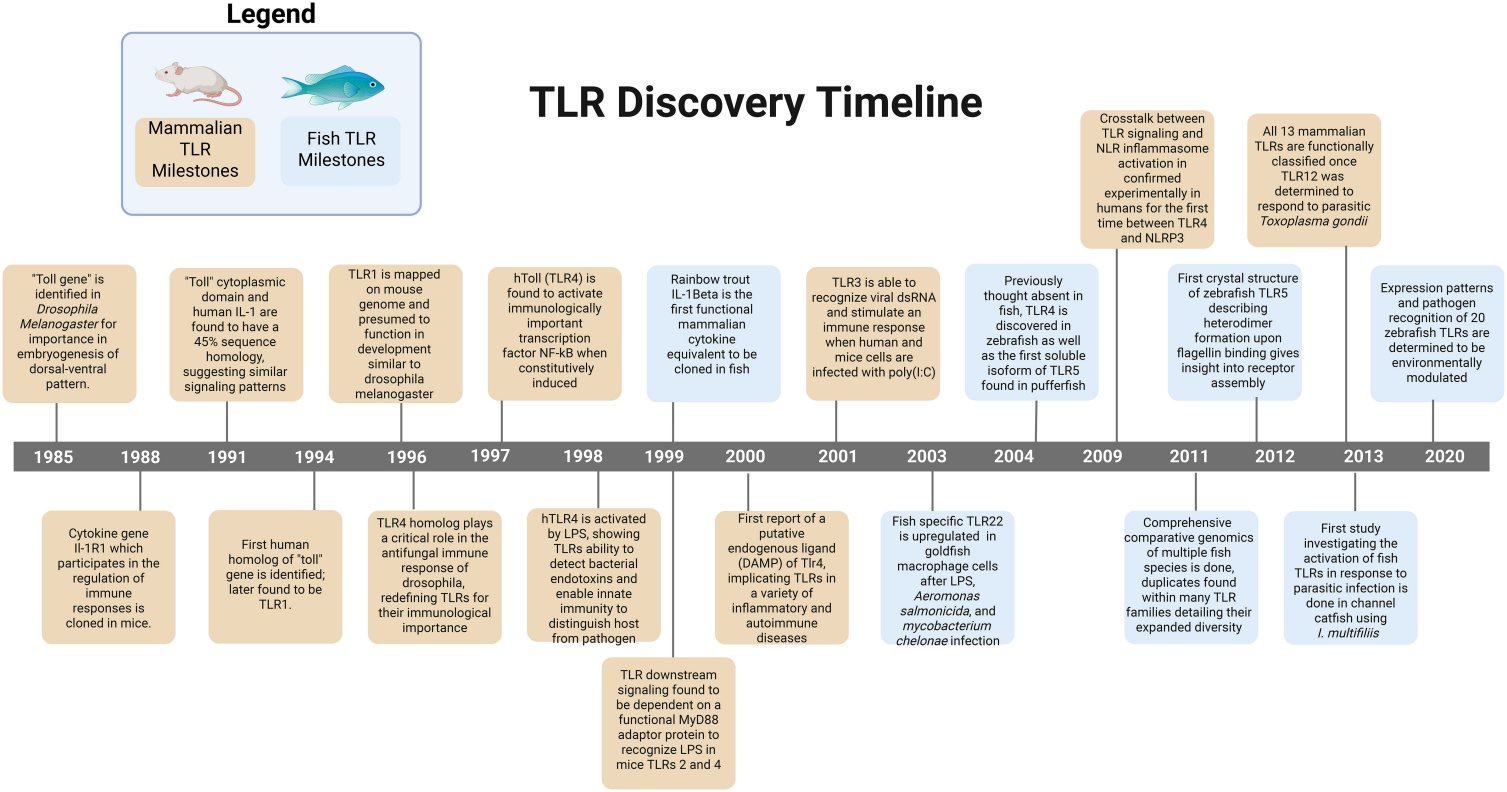
Timeline of TLR discovery in mammals and fish. This figure highlights the important discoveries and turning points in our understanding of mammalian (tan) and fish (blue) Toll-like receptors (TLRs), beginning with the foundational characterizations of basic TLR functionality, followed by studies that expanded our understanding of their role within specifically fish immune defenses by examining evolutionary and sequence trends, as well as ligand binding specificity. Although there is no doubt that other critical discoveries may be excluded for the sake of space, this timeline emphasizes the decades of work that have been done globally to bring about modern-day immunological applications of these receptors for human and fish medicine. Image created at BioRender.com.

“Das war ja toll!” or “that is amazing” were the words of German developmental geneticist Dr. Christiane Nüsslein-Vollhard in 1985 when her team of scientists identified the aptly named “Toll” gene as a critical component of *D. melanogaster* dorsal-ventral axis embryogenesis patterning (30). This was a significant breakthrough in invertebrate biology that, unbeknownst to the developmental biologists of the time, would serve as the first step in transforming our understanding of these proteins across vertebrate immunology. **Figure 1** provides a general summary of the major discoveries discussed in this section alongside other significant milestones.

Beyond the classification of “Toll” in embryogenesis, our understanding of the modern TLR and its associated pathways began to take shape with the cloning of the human Interleukin-1 receptor type 1 (IL-1R1) gene in 1988: an important inflammatory regulator implicated in pathways belonging to both the innate and adaptive immune systems that was deciphered before the TLR signaling pathways (31). Though it would take several years to make the direct connection between the “Toll” gene as belonging to this Toll/IL-1R (TIR) superfamily of immune receptors, the discovery was important for implicating a connection between the Drosophila “Toll” protein’s cytoplasmic domain and the previously cloned human IL-1 receptor which was found to contain 45% sequence homology in 1991 (32). The original conclusion drawn from this finding was that the Toll signaling pathway must regulate the localization of the dorsal-ventral axis during Drosophila embryonic development in a similar fashion to that of IL-1 receptor signaling (32). Of course, this wasn’t an exhaustive understanding of TLR functionality within vertebrates, but the knowledge of their shared ancestry would lead researchers to eventually investigate the potential of TLRs to activate inflammatory cytokines through interactions with the adaptor proteins in their TIR domain they shared with IL-1R, detailing the ancient role these receptors have played across various organisms in the broader TIR family (33).

In 1994, the first human homolog of the *D. melanogaster* toll receptor gene was identified among a cohort of 40 previously unidentified genes, but was not further investigated beyond a comparison of their transmembrane domain sequences (34). Researchers built upon this finding in 1996 by speculating on the potential role of another human gene encoding a toll protein known as Toll/interleukin-1 receptor-like (TIL), now known as TLR1, in human embryogenesis due to its sequence homology with Drosophila “Toll” and functional similarity to the mammalian IL-1 receptor (27). This assumption, although not inaccurate, would expand once the immunological role of the Drosophila “Toll” gene was revealed that same year.

Nobel Prize winner Jules A. Hoffmann and his team were the first to extend our perception of the Toll gene beyond a developmental biology context in 1996, revealing the immunological importance of “TI” (reclassified in 1997 as TLR 4) in the Drosophila antifungal response. By observing how the previously classified dorsoventral genes were induced upon immune challenge with *Aspergillus fumigatus*, it was concluded that the Toll genes have a greater role in adult drosophila innate immunity, with their involvement producing antimicrobial peptides crucial for full antimicrobial defense (35).

Following this discovery that shifted our understanding of TLRs from developmental biology to immunology, research sought to investigate if this mechanism was conserved within humans as well (36). In 1997, researchers described how hToll activated NF-κB activation and NF-ĸB controlled genes identically to dToll when constitutively activated, inducing the expression of inflammatory cytokines like IL-1 and IL-8, as well as initiating pathways leading to the expression of inducible nitric oxide synthase (iNOS) (36). This discovery was the first case of the Toll gene’s immunological role being explored in vertebrates and would serve as a pivotal moment in infectious disease research, with the knowledge that TLRs are conserved and essential across a wide range of phyla for proper immune signaling and function. Fellow Nobel Prize winner Bruce Beutler would expand upon these findings to decipher just how mammalian TLRs can activate these inflammatory cytokines with his research on the bacterial outer-cell membrane component, LPS, which is sensed by the mammalian endotoxin receptor TLR 4 (18, 37). Beutler found that, alongside TLR4, Myeloid differentiation factor-2 (MD-2) and cluster of differentiation 14 (CD-14) proteins found on monocyte and macrophage surfaces also bind LPS and associate with TLR4, highlighting the interconnected role of TLRs within the innate immune system to instantiate downstream signaling cascades (37).

TLR signaling was further investigated in 1999 when it was found that TLR2 and TLR4 in mice deficient in the MyD88 protein were unresponsive to stimulation by the LPS endotoxin, proving the importance of the adaptor protein for the activation of TLR4 immunostimulatory effects, specifically in response to LPS sensing (38). It is modernly known that MyD88 is involved in several other TLR signaling pathways besides those belonging to TLR family 3 and 4, but the discovery of the mechanisms by which TLRs signal entirely independently of MyD88 wouldn’t come until four years later. Beyond the microbial recognition of exogenous PAMPs like LPS, our understanding of TLRs within the immune system was once again shifted when they were found capable of binding putative endogenous damage-associated molecular pattern (DAMP) molecules. Ohashi et al. (2000) documented the earliest case within a mutant mouse isoform of TLR4 that was bound and elicited its pro-inflammatory activation by human DAMP heat shock protein 60 (hsp60), which implicated TLRs within the systems of immune discrimination between normal and stressed/damaged cells (39). In 2000, returning to the TIR domain, its crystal structure was examined by Xu et al. (2000) using mutagenesis and functional experimentation, providing a molecular foundation for uncovering the mechanism and complexity of TLR signal transduction (40).

By 2001, the first TLR capable of sensing non-bacterial nucleic acid antigens was characterized in murine TLR3, which responds to synthetic viral double-stranded RNA (dsRNA) analogue polyinosinic-polycytidylic acid (poly I:C) (41). Although it was previously known that poly I:C activated the transcription of a variety of pro-inflammatory cytokines and that protein kinase PKR was able to bind it, the specific mechanisms by which dsRNA was able to stimulate the immune system were largely unknown, and earlier research revealed the likelihood of a secondary receptor, that would be determined to be TLR3 (41). As mentioned earlier, the MyD88-independent pathway or TRIF-dependent pathway was uncovered in 2003 by contributions of several researchers that year (42, 43). TLR4 is determined to signal through both the TRIF-dependent and MyD88-dependent pathways, whereas TLR3 signals exclusively through the TRIF-dependent pathway. Similarly, most fish TLRs signal through the MyD88-dependent pathways. However, due to their expanded repertoire of TLRs, several species possess additional receptors able to signal through the TRIF-dependent pathways, reflecting an expansion in antiviral and more complex pathogen responses in fish immunity.

The first crystal structure of the extracellular LRR region in TLR3 was identified in 2005, providing a more complete image of the full TLR protein. This finding unveiled insights into its homodimer-forming nature and affinity for dsRNA (44).

A 2009 study on the activation of a related class of PRRs known as NLRs revealed that TLRs are involved in crosstalk and “priming” of these NLR proteins and their downstream signaling (45). Researchers found that assembly of the protein NLR family, Pyrin domain containing 3 (NLRP3) receptor’s inflammasome under LPS stimulation was prevented by the knockdown of the TLR4 gene in mice, inhibiting the expression of cytokine IL-1β (45). This was one of the very first documented cases of crosstalk between PRR signaling pathways, which unveiled their importance in the coregulation of inflammatory pathways, further implicating them as a multifaceted member within the PRR family (45).

The basic functionality of all mammalian TLRs, twelve in mice (*Mus musculus*) (TLRs1–9, 11-13) and ten in humans (TLRs1-10), was known by the year 2013 with the characterization of the previously unidentified TLR12 in mice as recognizing the protozoan parasite *Toxoplasma gondii* as well as regulating the production of cytokine IL-12; a function conserved with TLR11 in mice that corresponds with their subsequent heterodimer forming nature (46). NMR spectroscopy data revealed in 2014 the structure of the remaining TLR domain to be modeled in the TM region that provided biochemical data into TLR3’s affinity for dimerization due to helix-helix interaction affinity, presenting valuable information into the signal transduction of TLR3 (47).

In contrast, our knowledge of teleost TLRs remains comparatively limited, though interest has grown substantially alongside the expansion of global aquaculture.

#### 2.2.2 Discovery in fish

Despite the comparatively slower pace of fish-specific TLR discovery relative to their mammalian counterparts, the rapid expansion of global aquaculture in the past few decades has increased research interest in teleost immunology. Using existing mammalian knowledge and a comparative immunology approach, researchers identified in fish an expanded repertoire of innate immune defenses, mediated by a distinct set of TLRs not found in mammalian systems. We can begin documenting the TLR discoveries in fish by starting similarly to mammals with the molecular cloning of rainbow trout (*Oncorhynchus mykiss*) cytokine interleukin 1 beta (IL-1β) receptor, which was completed in 1999 (48). The significance of this finding, beyond reaffirming that fish IL-1Rs and TLRs have a conserved TIR domain similar to mammals, was that this was the first example of a non-mammalian IL-1 sequence to be published, serving as a major step forward in establishing the link between the innate and adaptive immune systems in fish, as it was just beginning to be understood in mammals (51). Only a year after Beutler published his findings of the upregulation of mouse TLR 4 in response to LPS, James L. Stafford and his team in 2003 published their observations of a TLR gene upregulation in goldfish macrophage cells when exposed to bacterial LPS, heat-killed *Aeromonas salmonicida*, and live *Mycobacterium chelonei* (49). This was the first TLR classified within the teleost family and would later be designated as TLR22, a fish-specific TLR that plays a significant role in mucosal and systemic defenses against both bacterial and viral infections (50). The discovery of novel fish TLRs is currently ongoing, with up to 22 TLRs identified in some teleost species, thus far, and research to better understand, identify, and target these receptors is currently ongoing (51).

The existence of TLRs lacking direct mammalian homologs was an important finding for our understanding of fish immunology that revealed a more complex and expanded innate immune system in fish than was previously thought of these lower vertebrate species. This knowledge would provide insights into fish disease resistance that would serve as the foundation for the development of new drugs and vaccines for these identified therapeutic targets that have been more recently explored in the past 1–2 decades. In 2004, many fish-specific TLRs were identified by several researchers. The classification of a duplicate zebrafish TLR4 isoform, TLR18, TLR19, two TLR20 isoforms (TLR20a/b), TLR 21, and TLR22 by Meijer et al. (2004) established an ancient common ancestor for all vertebrate TLRs, disproving that there was a more recent common mammalian ancestral gene (52).

**Figure 2:**
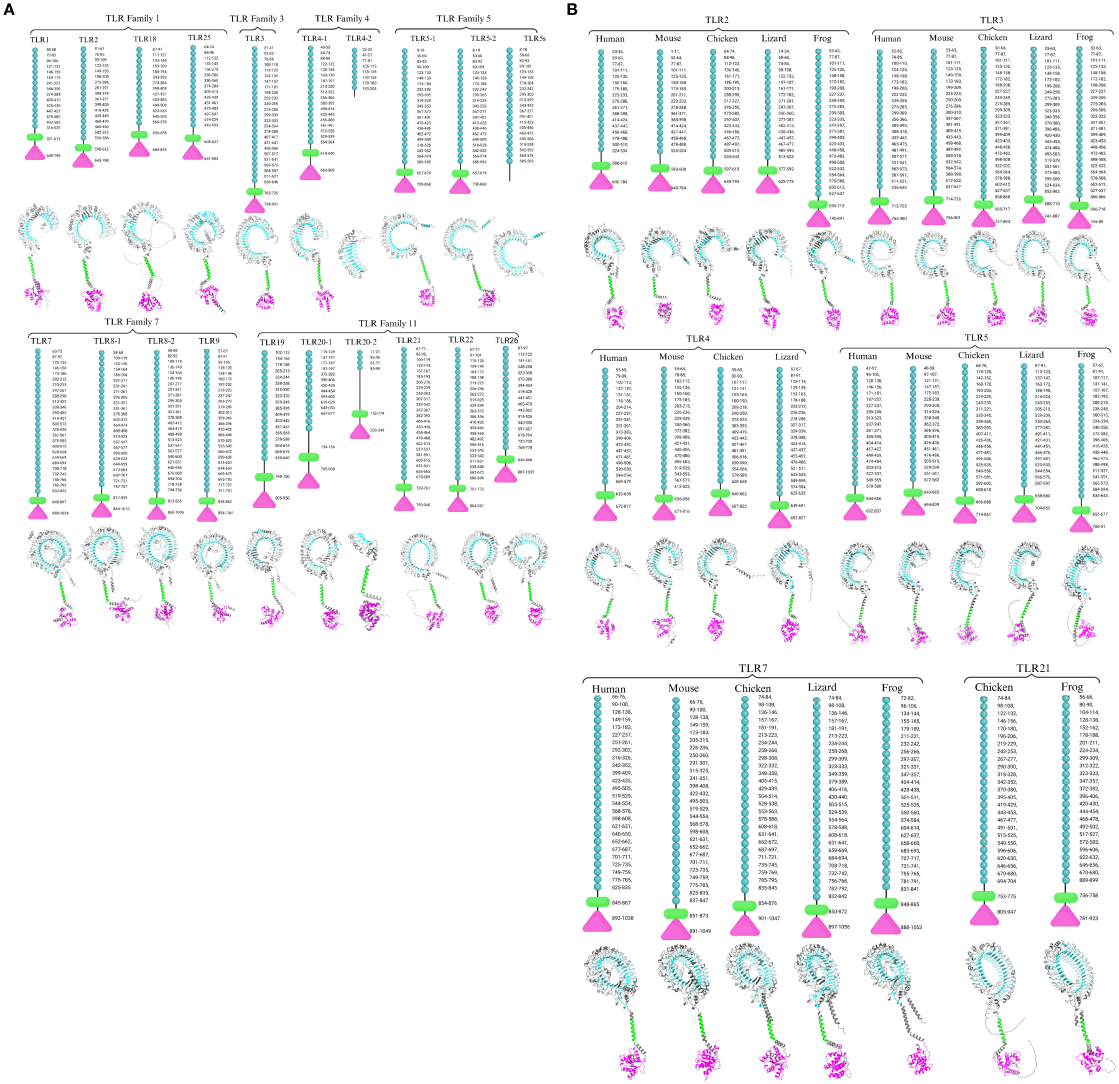
Comparative view of 2D and 3D Structures of TLR Variants. **(A)** Channel Catfish TLR Families and Variants. Multiple TLR families and their variants are shown in channel catfish to be representative of fish as a whole and to serve as a model to compare to their non-fish counterparts. **(B)** Comparative Mammal, Avian, and Reptile TLR Variants. TLR sequences used include human (*Homo sapiens*), mouse (*Mus musculus*), chicken (G*allus gallus*), green anole (*Anolis carolinensis*), and western clawed frog (*Xenopus tropicali*s). Only TLRs with clear orthologs in channel catfish (TLR2, TLR3, TLR4, TLR5, TLR7, and TLR21) were included for cross-species comparison. Domain organization and predicted structures are largely conserved across species, with minor variation in LRR number and spacing reflecting species-specific adaptation. Leucine-rich repeats (LRR) (teal), transmembrane (TM) (green), and toll/interleukin-1 receptor domain (TIR) (pink) are highlighted to describe structural variations within and between families. LRRs are present in each TLR due to their direct interaction with ligands, whereas TM and TIR regions may not be present in certain isoforms due to localization. Proteins are displayed in 2D (top) and 3D (bottom) representations from N-terminus (top of protein) to C-terminus (bottom of protein). The LRR region was determined using LRRsearch.com, TIR and TM regions were determined using SMART software, 3-D models were predicted by AlphaFold Server, and colored using ChimeraX. 2-D models and final image formatting were created in Biorender.com (72).

As interest in finding ways to modulate this expanded innate immune system of fish to enhance disease management in aquaculture grows, recent studies have focused on TLR functionality/ligand binding as well as computational and phylogenetic analysis. One of the first of these was a 2011 study investigating the comprehensive comparative genomics of multiple fish species that led to the assessment of multiple duplicates across many TLR families, contributing to the expanded diversity of their functionality as pathogen recognition systems, an adaptive trait necessary for their free-flowing environment (53). X-ray crystallography modeling of many human TLRs was completed in the late 2000s and provided insights into the mechanisms by which TLRs activate and bind ligands when coupled with earlier experimental, immunological, and biochemical data (54). The first fish TLR to be imaged using x-ray crystallography was in zebrafish TLR5, which revealed a detailed mechanism for heterodimer formation upon *Salmonella* flagellin binding in 2012 (55). This study confirmed the conservation of TLR5 signaling between mammals and fish, as well as exemplifying how extensive site-specific mutagenesis and phylogenetic comparisons revealed a great deal of early information about the functionality of these immunologically important receptors beyond model mammalian species. New forms of computational modeling in the past decade and the introduction of the protein prediction software and database, AlphaFold, in the late 2010s, rapidly enriched a variety of immunological research fields, allowing for the rendering of full-length hypothetical 3-D TLR structures, refining previous models, as well as facilitating visual and sequence comparisons of domain architecture as illustrated by **Figure 2A** (56).

The early TLR findings that have been done in higher vertebrate species certainly informed many aspects of the following fish-specific discoveries; however, the increasing overlap of human and veterinary medicine has created an opportunity for the reciprocal exchange of important immunological knowledge. These fields inform the development of advanced therapeutics founded in the shared understanding of their immune mechanisms, diversity, and evolutionary history.

## 3 Structure, overview of TLR families, and evolution

### 3.1 Structural domain classification of TLRs

As indicated above, most TLRs consist of three main domains: the LRR, TM, and TIR domains from N to C terminus, respectively, which can be identified in **Figure 2A**. The LRR region is located extracellularly, known as the ectodomain (ECD), whereas the TIR domain is located intracellularly, separated by a TM domain that spans the membrane once (57). TLRs lacking this TM domain exist in the soluble form, found intracellularly (58).

Regarding the structure of TLRs, the ECD consists of tandem LRR motifs, which provide the structural basis for pathogen recognition in a wide range of organisms (59). Typically, each LRR motif consists of 20–29 amino acids and encompasses a consensus sequence such as xLxxLxLxxNxLxxLxxxxFxxLx, where L is reserved for hydrophobic residues, which are usually represented by leucine, isoleucine, valine, or phenylalanine; N represents a conserved polar residue that could be asparagine, threonine, serine, or cysteine; F is the amino acid phenylalanine; and x is any amino acid (25, 60, 61). Such repeats fold into a curved, horseshoe-shaped solenoid structure, with a β-sheet lining the concave surface while the convex side is made up of variable loops or helical elements (62). In fish TLRs, like their mammalian counterparts, ligand binding often occurs on the concave or ascending lateral surface of the LRR region, where N-linked glycans are absent and thus direct molecular interaction with ligands can occur (61). The domain dictates not only the orientation of the ligand but also favors dimerization with the TM domain of TLR-bound ligands upon engagement. These binding interactions can be visualized in detail in Section 5, displaying examples of docking structures. The LRR domain is also conserved across other PRRs, including NLRs, RLRs, and CLRs, making it vital for pathogen sensing and innate immunity (63, 64). It is worth mentioning that there are significant limitations when defining the LRRs of the extracellular domain using prediction tools due to the presence of non-consensus sequences and variations in the criteria for defining LRRs across different models (65).

TLRs have a TM domain, serving not only as a membrane anchor but also as an active contributor to the receptor assembly (25). TM domains can associate independently from both the ECD and the intracellular TIR domain and do so with definite specificity since they do not interact randomly with unrelated transmembrane helices, allowing isoforms, closely related homologs, and identical TLRs to dimerize (66). Such TM interactions are important for the stabilization of preassembled receptor dimers before the engagement of the ligand, particularly for the heterodimerization of homologs such as TLR5–1 and TLR5–2 displayed in Section 5 (66, 67). Importantly, TM domains possess intrinsic oligomerization potential that needs to be restrained to avoid constitutive signaling and is unleashed by ligand binding. Many TLR TM helices, in particular those of TLR2, TLR7, and TLR9, contain conserved Small-xxx-Small motifs (small residues are typically Gly, Ala, or Ser), a well-established transmembrane helix–helix packing motif, analogous to GxxxG, which is known to cause oligomerization in membranes (66, 67). These properties together position the TM domain as a key regulatory element in TLR activation and as an underexplored therapeutic target, with potential relevance for the design of vaccine adjuvants that modulate receptor assembly other than just ligand recognition alone.

The intracellular TIR domain forms the conserved signaling core of fish TLRs and couples ligand-induced receptor dimerization to downstream immune activation through homotypic TIR-TIR interactions with adaptor molecules such as MyD88 and TRIF (68–70). Sequence analysis across the teleosts indicates that, while divergence of surrounding regions occurs, the fish TIR domains retain the canonical Box 1 ((F/Y)DAΦΦXΦ motif for β-sheet), Box 2 (signaling loop), Box 3 (FWXXΦ motif for α-helix) motifs required for signal transduction, and thus, there is strong evolutionary conservation of TIR-mediated signaling function (68). In fish, the TIR-domain-containing adaptor proteins are cytoplasmic and are selectively recruited upon receptor activation to drive NF-κB and type I interferon signaling, and both the MyD88 and TRIF-dependent pathways contribute to antiviral immune responses (69, 70). Notably, teleost TRIF lacks the N- and C-terminal proline-rich regions present in mammals, supporting the idea that TIR-domain signaling in fish depends more directly on conserved TIR–TIR interactions rather than auxiliary regulatory domains (70). In this regard, the conserved yet adaptable mechanisms mediated by TIR drive antiviral immunity in fish and define a key intracellular regulatory site (68, 69).

To illustrate domain organization among teleost TLRs, channel catfish (*Ictalurus punctatus*) TLRs were used as representative models in **Figure 2A**. Access codes for catfish genome sequences were sourced from the Quiniou et al. (2013) study detailing a comprehensive characterization of channel catfish TLRs (8). The LRR, TM, and TIR regions were identified using standard domain-prediction tools and visualized in both two- and three-dimensional formats to highlight conserved structural features and family-specific variation.

In channel catfish, TLR4-2 (TLR4s) and TLR5S lack both the transmembrane and TIR domains, indicating that they exist as soluble receptors, which correlates with channel catfish studies done *in vivo* (8, 58). Surprisingly, TLR4b in zebrafish remains insoluble with a TM and TIR domain, indicating further gene expansion that varies across teleost species. Also, TLR20–2 lacks key ligand-binding residues, possibly acting as a pseudogene (71). This aligns with observations of TLR20–1 in grass carp (*Ctenopharyngodon idella*), which does not express upon pathogen stimulation (8, 71). These assumptions have yet to be experimentally confirmed, but multiple papers have discussed the likelihood of their non-functionality.

### 3.2 Overview of fish TLR families

This section will provide a brief overview of all known TLRs across teleost species and within their respective TLR families, as well as making connections to their higher vertebrate counterparts to identify vital, conserved functional features. Beyond their immunological roles, TLRs have also evolved to be involved in mammalian tissue repair and regeneration, but we will be focusing specifically on their immunological roles as pathogen receptors (73). The six conserved TLR families are the TLR1, TLR3, TLR4, TLR5, TLR7, and TLR11 families. Although fish share many of the same TLRs with mammals, except for TLR 6, TLR10, and TLR12, some species have nearly doubled the number of mammalian isoforms. Despite sequence homologs of these TLRs being absent in fish, we include them for the purpose of examining species-specific distinctions and preserved mechanisms that may produce insights for receptor targeting. Due to their more recent classification, there is less understood about the functionality and signaling of these novel TLRs. By organizing TLRs into their respective families and examining their existence across diverse phylogenetic lineages, we provide a more comprehensive understanding of the evolutionary origins, species-specific functions, signaling pathways, and structural features of these receptors.

#### 3.2.1 TLR family 1

The TLR1 family is one of the most expanded, with up to 10 members between mammalian and teleost species. TLR family 1 includes: TLR1, TLR2, TLR6, TLR10, TLR14, TLR15, TLR18, TLR24, TLR25, & TLR27. TLR6 and TLR10 remain to be identified in fish; however, they are assessed in this section to identify functional homolog comparisons. The TLR family 1 has also proved to be quite diverse and species-specialized across phyla; however, their functionality remains in extracellularly recognizing lipopeptide PAMPs like synthetic lipopeptide analog Pam3CSK4 or gram-negative bacterial lipoproteins and eliciting the MyD88 dependent signaling pathway to induce the expression of inflammatory cytokines by transcription factor NF-κB (74).

TLR1 was the very first TLR to be identified in humans and has subsequently been discovered and characterized in a variety of fish including: Dabry’s sturgeon (*Sinosturio dabryanus*), Nile tilapia (*Oreochromis niloticus*), turbot (*Scophthalmus maximus*), rainbow trout, orange spotted grouper (*Epinephelus coioides*), large yellow croaker (*Larimichthys crocea*), miiuy croaker (*Miichthys miiuy*), golden pompano (*Trachinotus blochii*), yellow catfish (*Ameiurus natalis)*, crucian carp (*Carassius carassius*), zebrafish, largemouth bass (*Micropterus salmoides*), humpback grouper (*Cromileptes altivelis*), channel catfish, Chinese perch (*Siniperca chuatsi*), Asian swamp eel (*Monopterus albus*), common carp, blunt snout bream (*Megalobrama amblycephala*), and grass carp (75–87). Few absences of the TLR1 gene have been recorded across teleost species, as the structure remains highly conserved due to its important role in recognizing bacterial lipoproteins and triacylated lipopeptides, such as those associated with the peptidoglycan (PGN) layer of gram-negative bacteria. One exception, however, is Atlantic Cod (*Gadus morhua*), which has been recorded to lack TLR1 and other mammalian bacterial-sensing TLRs (88, 89). This loss of the higher vertebrate bacterial receptors suggests evolutionary pressure for compensation by fish-specific bacterial-sensing TLRs like TLR14 and TLR25 that may be more suited for their environment-specific pathogens (89). Another irregularity in TLR1 conservation is that both common carp (*Cyprinus carpio*) and rainbow trout contain duplicate TLR1 isoforms with one functional TLR1 gene and an additional truncated copy considered the “true” TLR1 ortholog that has lost its functionality throughout evolution and exists as a pseudogene today (90).

TLR1 in mammals and fish does not activate as a singular receptor but rather forms heterodimers upon PAMP stimulation with other TLR family 1 and possibly TLR family 11 receptors (92). In mammals, TLR1 is well established as cooperating with TLR2, TLR6, and TLR10 when bacterial lipopeptide, specifically triacylated lipopeptides with three fatty acid chains, are recognized, initiating binding by interactions in their TM and TIR domains to activate downstream signaling and inflammation (92). Pam3CSK4 is an example of a synthetic lipopeptide known to elicit the formation of the TLR1/2 heterodimer, but another small molecule, CU-T12-9, also selectively induces TLR1/2 association, activating NF-κB through the MyD88/tumor necrosis factor receptor-associated factor/Inhibitor of kappa-B kinase (TRAF/IKK) (91).

The structure of the actual TLR1 protein between fish and mammals is quite similar in that they are both found extracellularly anchored in the cellular membrane by the TM domain with a highly conserved TIR signaling domain, most likely influenced by purifying selection (92). However, there exists a significant amount of variation in the amino acid length of the LRR domain, with anywhere between 19 and 21 LRRs on average, and around 20–30 residues in length between mammals and fish (93). Japanese pufferfish and zebrafish have been noted to have one additional LRR as compared to mammalian TLR1, with 20 LRRs, whereas only around 15 LRRs were identified in channel catfish using LRRsearch.com, a tool specifically for identifying the LRR residues in protein structures (74, 93). There is ongoing debate as to the exact number of LRRs in TLRs across teleost species due to divergence from the typical mammalian model structure, which most tools are based on, that contributes to the misidentification of fish protein domains. Beyond residue count, there is also variation in the LRR sequencing motifs; for example, turbot TLR1’s cysteine clusters are positioned towards the N-terminal of their LRR domain (LRRNT), whereas Nile tilapia TLR1 has its LRRs positioned at their C-terminal LRR (LRRCT) (76). It is speculated that the LRRNT motif of Turbot could reveal a strong affiliation with TLR2 since mammalian TLR1 LRRNT is believed to be an essential structural component for the formation of the TLR1/TLR2 heterodimer that has been adopted as a true mechanism of TLR1 activation, although analyzing its specific formation *in vivo* remains an ongoing area of study in teleost (74).

TLR2 is also a member of the TLR family 1. It is similarly well conserved across all mammals and most teleost species in a similar fashion to TLR1, including but not limited to: zebrafish, Japanese flounder (*Paralichthys olivaceus*), channel catfish, grouper (*Epinephelidae*), Antarctic fish (*Notothenioidei*), miiuy croaker, grass carp, common carp, golden pompano, Dabry’s sturgeon, and rainbow trout (94, 95). TLR2 is ubiquitous between mammals and fish, once again apart from Atlantic salmon, which fully lack any functional or non-functional TLR2 genes (88). Some variations exist in their specific amino acid sequences, but their domain architecture, function, and downstream signaling remain consistent between phyla. The only significant deviation from this case is the existence of predominant gene duplication in many fish as well as some bird, mammal, and reptile species (95, 96). Whereas the TLR2 duplicates in higher vertebrates like humans and dogs are considered pseudogenes, TLR2a and TLR2b are two functional isoforms expressed in common carp and rainbow trout species that share a majority of their amino acid identities and can form heterodimers with TLRs 1 and 4 upon stimulation with lipopeptide ligands (97).

The extracellular binding domain of TLR2 varies somewhat, with mammals possessing around 18–20 LRRs and fish species possessing anywhere from 7 LRRs in yellow catfish to 23 LRRs in rohu and other Indian Carp species (25, 98). TLR2a/b activation and signaling are fully dependent on the presence of MyD88 in fish, as well as higher vertebrates, where it has expanded to form homodimers with mammalian TLR6 and TLR10. Not all fish species have these duplicate TLR2 isoforms, as is shown in **Figure 2A** by the presence of only one TLR2 protein in the channel catfish genome. Their role in recognizing the archetypal ligands of Lipoteichoic acid (LTA), a key antigenic component of cell surface gram-positive bacteria, PGN, and Pam3CSK4, remains conserved between higher and lower vertebrates in agreement with the rest of the TLR family 1 receptors (53, 99). TLR2 in fish has been recognized for its importance in responding primarily to a variety of aquatic bacterial pathogens, as well as participating in the apoptotic machinery across different species, including hybrid yellow catfish (100). This phenomenon is especially important for considering how this functionality of TLR2 as a “death receptor” can be leveraged for developing effective immune therapies that enhance pathogen resistance through this alternative pathway (100). TLR2 in mammals has been generally accepted as capable of forming homodimers under ligand recognition and receptor association (101–103). This evidence has yet to be replicated in teleost species, but considering the extent of TLR2 feature conservation, it is likely that this function is also preserved in fish.

To briefly discuss TLR6 and TLR10, they are considered mammalian-specific, and consequently, both are absent in all teleost species (25). In mice, TLR10 is a nonfunctional pseudogene, an unfortunate fact for their use as immunological models, seeing as TLR6 and TLR10 are both present and functional within human pathogen recognition (104). Their ligand-recognition capabilities have not been lost in fish, however, as it is theorized that another member of the TLR family 1, TLR14, is a fish-specific analog that may have recovered their functionality, as we will discuss in further depth in the following section (105). The ligands that induce TLR1 and TLR2 are also capable of activating TLR10 due to their heterodimer-forming relationship upon recognition of PAMPs such as diacylated lipopeptides Pam3Cys and FSL-1 that are associated with Lyme disease caused by *Borrelia burgdorferi* infection (106). Curiously, TLR10 has also been recognized as forming homodimers upon stimulation with immunosuppressant 1,25-dihydroxyvitamin D_3,_ downregulating TLR2, 4, and 5, suggesting its ability to function independently of distinct receptors (107). TLR10 is thus considered a broad TLR signaling suppressant of the mammal immune system, regulating inflammation through interference with both the MyD88-dependent and TRIF pathways (108).

TLR14 is a fish-specific, mainly intracellular receptor that has also been revealed to co-localize in the cell membrane upon recognition of Gram-positive and Gram-negative bacterial ligands like LPS and flagellin (109). TLR14 has been studied in select species, including Asian swamp eel, Japanese flounder, miiuy croaker, golden pompano, Mandarin fish (*Synchiropus splendidus*), sea lamprey (*Petromyzon marinus*), and Atlantic cod (105, 109, 110). However, TLR14 is absent in several fish species, such as zebrafish and channel catfish, as seen by their lack of TLR14 protein in **Figure 2A**. There has been some confusion around the classification and nomenclature surrounding TLR14, leading to its misidentification early on in some fish species and eventual reclassification as TLR18, a functional homolog that has “recovered” the function of TLR14 in those species lacking it; a fact we will dive further into in later sections (29). The number of LRRs in TLR14 is highly conserved across species, with between 5 and 8 LRR domains across various fish species (109). TLR14 is MyD88-dependent and has been implicated in various studies for viral and bacterial defense due to its upregulation upon stimulation with poly I:C, flagellin, and LPS, where it induces the NF-κB and MAPK pathways capable of expressing pro-inflammatory cytokines like TNF-*α* and IL-6 (109, 111).

TLR18 is another fish-specific receptor in TLR family 1 that has been reported in a variety of fish species, including zebrafish, Atlantic salmon (*Salmo salar*), grass carp, yellow catfish, common carp, so-iuy mullet (*Liza haematocheila*), and Nile tilapia (77, 112). The conserved structure of so-iuy mullet TLR18 has been modeled as an open-looped horseshoe-shaped structure with 19 parallel beta strands of LRR repeats 1–19 forming a concave binding region that is also capable of potentially forming homodimers at LRRs 10-14. TLR18 functions through the MyD88-dependent pathway, where it activates NF-κB signaling in grass carp and common carp cells when over-expressed, inducing the expression of TNF-*α*, IFNs, IL-1β, IL-8, and IL-10 (113, 114). The specific functionality of TLR18 has been implicated in a variety of bacterial and viral sensing applications with infectious salmon anemia virus (ISAV), a ssRNA-based virus, infection failing to upregulate TLR18 expression, whereas poly I:C treatment, a synthetic dsRNA, has been documented to upregulate TLR18 expression in Nile tilapia, as well as potentially PGN and LPS. Grass carp reovirus (GCRV), a dsRNA virus, and *Aeromonas hydrophila* inoculation have also been found to stimulate TLR18 in grass carp, illustrating the diversity in the ligand-specificity of TLR18 across various fish species (77, 113).

A following fish-specific TLR is TLR24, which is unique in that it is only found in jawless fish such as lamprey species and is completely absent in bony fish and higher vertebrate species. Although previously classified in their own families, through computational methods to examine strand specificity, TLR24 was classified into the TLR1 subfamily (115). The sea lamprey genome contains four copies of extracellular TLR24 (TLR24a-d), which are most closely related to TLR2 and functional, except TLR24c, which has an early stop codon, suggesting it is a potential pseudogene. In Japanese lamprey stimulated with poly I:C, TLR24a was downregulated 6 hours post-injection, whereas other TLR24 genes were upregulated and therefore potentially poly I:C inducible and controlled by extracellular microbial PAMPs. Due to the limited number of studies done on this novel TLR, the LRR count and overall domain architecture of TLR24 remain poorly determined. However, one lamprey study using the SMART domain-prediction software predicted approximately 3–6 LRRs within the extracellular domain (116).

TLR25 is a fish-specific sensor that plays an important role in the innate defenses against viral and bacterial infections, while the precise nature of its ligand has yet to be identified. It is localized intracellularly and trafficked by chaperone proteins from the endoplasmic reticulum to signal via the MyD88 pathway, inducing both the NF-κB and type 1 IFN signaling responses (117, 118). TLR25 functionality tends to vary between specific lineages such as how overexpression of Ya-fish (*Schizothorax prenanti*) TLR25 upregulates NF-κB downstream inflammation in HEK293T cells, but Nile tilapia TLR25 are unable to do the same, potentially suggesting they are incapable of recognizing the canonical mammalian adaptor proteins of MyD88 or TRIF, or that TLR25 in Nile tilapia requires the formation of distinct heterodimers or certain adaptor molecules (119). Nonetheless, TLR25 signaling in Ya-fish has been evaluated to promote transcription factor IRF1 expression of IFNα selectively (118). The number of LRRs of TLR25 are documented to be somewhat consistent across fish species with most teleost possessing around twenty LRR motifs. TLR25 is fairly conserved across fish, with certain exceptions: zebrafish lack TLR25 completely, whereas Atlantic cod possess an expanded repertoire of seven TLR25 duplicates (88). TLR25 is also present in channel catfish, spotted gar (*Lepisosteus oculatus*), common carp, and grass carp (88, 119).

#### 3.2.2 TLR family 3

The TLR family 3 contains a single member: TLR3, which is a highly conserved gene that has not undergone duplication in mammals or birds and scarcely in teleosts, with the exception of fish like crucian carp (96, 120). TLR3 is functionally considered a viral-sensing TLR for its ability to bind dsRNAs, but it also binds endogenous cellular mRNA and sequence-independent small interfering RNA (121). TLR3 is within the group of endogenous TLRs found within the endosomal and lysosomal membranes, but unlike most TLRs, TLR3 signals through the TRIF-dependent pathway rather than the MyD88-dependent pathway, where it initiates a strong antiviral response, releasing IFNα, IFNβ and pro-inflammatory cytokines like IL-6 by the expression of transcription factors NF-κB and IRF3 (122, 123). For activation of TLR3, as well as other endosomal TLRs, cleavage by endosomal proteases is required to stabilize the TLR3/3 dimer and allow for ligand recognition to occur (124). Structural variations across fish species remain modest due to conservation of domain architecture with higher vertebrates, with the number of LRRs in TLR3 ranging between 24 LRRs in zebrafish and 27 LRRs in Indian carp. Functional binding studies have determined that poly I:C interacts with fish TLRs between LRRs 2–3 and LRRs 19-28 (121). TLR3 is widely distributed across various teleost fishes, and species-specific polymorphisms exist, such as in grass carp TLR3, which has been investigated for its importance as a receptor of dsRNA triggering downstream signaling that has resulted in resistance to GCRV, offering potential breeding targets for disease-resistant species. Alternatively, hybrid yellow catfish TLR3 has been observed to upregulate significantly upon sensing of *Aeromonas hydrophila*, demonstrating yet another deviation of TLR3 functionality since a similar treatment with gram-positive bacteria fails to induce the same response in zebrafish TLR3 and only retains some upregulation upon gram-negative sensing (125). This phenomenon, as well as TLR3’s limited duplication across lineages, highlights the central role that TLR3 plays in the antiviral response across higher and lower vertebrates that has resulted in its purifying selection across lineages.

#### 3.2.3 TLR family 4

TLR family 4 remains the only TLR family not ubiquitously found across all vertebrates. Within higher vertebrate lineages, TLR4 is the sole member of TLR family 4. Fish fail to consistently follow these patterns of conservation with varying levels of functionality and isoform number across different species, suggesting that teleost TLR4 functions more as a paralog than a direct ortholog of mammalian TLR4 (126). In mammals, TLR4 is characterized by its ability to recognize LPS, a main structural component and endotoxin of gram-negative bacteria that binds to form the TLR4/4 homodimer. When it is expressed in fish, TLR4 typically has lower sensitivity to LPS, requiring extremely high levels of the PAMP to activate, while retaining the ability to recognize heat shock proteins; a function also observed in its mammalian homolog (127, 128). One exception is TLR4 in blunt snout bream that possesses a fully functional homolog of mammalian TLR4 capable of responding to bacterial infection (129). The number and structure of TLR4 copies vary across fish species, such as in zebrafish that possess two TLR4 copies, known as TLR4a and TLR4b, common carp, which possess three functional copies, including TLR4-1, TLR4-2, and TLR4-3, as well as channel catfish that possess two TLR4 isoforms, TLR4–1 and TLR4-2 (99, 128). There are also clear distinctions in the accessory proteins necessary for effective signaling cascades since mammalian TLR4 utilizes myeloid differentiation factor 2 (MD-2) and CD14 to trigger inflammation, whereas fish TLR4 signaling is often done without MD-2 and CD14 since many fish species lack the accessory protein and TLR4 coding genes completely (130). A gene encoding an MD-2–like protein has been recently identified in zebrafish. However, Loes et al. (2021) determined by *in vitro* testing of zebrafish TLR4a and TLR4b that the TLR4/MD-2 complex is unable to effectively respond to LPS suggesting that teleost TLR4/MD-2 sensitivity to LPS may be an ancestral feature found more often in higher tetrapod vertebrates that maintains lower levels of functionality among certain fish species and implies that there could potentially be other canonical ligands for TLR4 activation in fish (130). The localization of TLR4 remains conserved in fish, with its expression on the surface of immune cells as well as its association with MyD88 for downstream signaling to induce the expression of pro-inflammatory cytokines in higher and lower vertebrates. One exception is channel catfish TLR4-2, which is considered a putative soluble TLR4 gene, as illustrated in **Figure 2A** by the lack of a TM and TIR domain, that potentially acts as a negative regulator of immune activation (8). There is significant variation in the LRR region of TLR4 across fish species, reflecting lineage-specific adaptations of its structural components to the diverse environmental and pathogenic pressures throughout the evolutionary history of teleost fish (131). Additionally, TLR4 has been identified in grass carp, Chinese Rare Minnow (*Gobiocypris*), Wuchang bream (*Megalobrama amblycephala*), and rohu (131).

#### 3.2.4 TLR family 5

TLR5 is the singular member of the TLR family 5 and is conserved between higher and lower vertebrates, with several duplications across teleost lineages. TLR5 functionality is also consistent across phylogenies, primarily detecting bacterial flagellin, inducing homodimerization in mammalian species to upregulate inflammatory responses through the MyD88-dependent pathway (132, 133). However, this is not the same for many fish species, such as zebrafish, which possess two distinct copies of TLR5 (TLR5a and TLR5b) that form a heterodimer complex that must both be expressed together to activate the NF-κB mediated pathway and the associated pro-inflammatory cytokines upon flagellin stimulation (134). TLR5 is located on the cell surface of immune and epithelial cells of all vertebrates, with certain fish also possessing soluble isoforms of TLR5 lacking the TM and TIR domains, including Japanese pufferfish, rainbow trout, and channel catfish, as illustrated in **Figure 2A** (135). These soluble isoforms function by diffusing into the extracellular matrix where they bind TLR5 ligands, acting as negative regulators of inflammation. This unique case is important to consider since TLRs are most commonly positive regulators of inflammation especially in mammals, where they are often studied in autoimmune research for means to negatively regulate and control their expression (5, 136). These soluble isoforms have been investigated in trout by Wangkahart et al. (2018), who found distinct tissue expressions between the membrane and soluble forms of TLR5, observing competition by TLR5s for flagellin binding. These findings imply TLR5s as a putative negative regulator of TLR5M-induced inflammation across fish lineages (137). Mammalian TLR5 typically possesses around twenty-two LRR motifs, which is somewhat conserved with fish, considering instances of species-specific variations of TLR5M and TLR5s between lineages, such as mrigal possessing 16 LRRs, whereas orange spotted grouper and zebrafish possess twenty-three and twenty-two LRR motifs, respectively (93, 134, 138, 139). The binding of channel catfish TLR5–1 and TLR5–2 to *Edwardsiella piscicida* flagellin is further detailed in Section 5.1, highlighting key LRRs and exemplifying the formation of the TLR5 heterodimer that assembles across teleost species. TLR5 is highly conserved within fish species, except for Atlantic cod and lamprey, which completely lack members of the TLR family 5. In the case of Atlantic cod, Solbakken et al. (2016) suggested that this loss of TLR5 is likely driven by genetic drift and different environmental pressures that have created the unique case of its large gene expansion of Major Histocompatibility Complex Class-1 (MHC-1) but losses of several TLRs and the duplication of individual paralogs like TLR 7, 8, 9, 22, and 25 (88). Recent studies in Cyprinid species revealed that duplicated TLR5 isoforms in grass carp are activated by dsRNA stimulation upon recognition by the TLR5a/b dimer. This finding reveals that TLR5 variants in fish have novel functional roles beyond traditional flagellin sensing, contributing to pathways such as the antiviral response (140). These discoveries expand our current understanding of PAMP recognition diversification within the teleost TLR family 5.

#### 3.2.5 TLR family 7

The TLR family 7 contains TLR7, TLR8, and TLR9, which are involved primarily in the response to microbial nucleic acids released by various bacterial and viral sources. These receptors are localized within endosomal membranes where they recognize RNA/DNA molecules and motifs released following engulfment by immune and epithelial cells (124). Ligand binding occurs once proteolytic cleavage of their ectodomains occurs, activating the TLRs and inducing Th-1 type immune responses (141, 142). This triggers a strong antiviral immune response through the release of pro-inflammatory cytokines by the MyD88-dependent pathways that primarily upregulate inflammation in fish but have also been observed to downregulate mammalian inflammatory responses (143). Members of the TLR family 7 are found in all vertebrate species, with lineage-specific exceptions in some avian species, like chicken, which have completely lost the TLR9 gene and lost functionality of TLR8, as well as jawless fish species like lamprey that lack TLR9 altogether (144, 145). Members of the TLR family 7 are favorable targets for adjuvant research in fish because they offer broad pathogen recognition and protection that enhances especially mucosal immunity (146).

TLR7 and TLR8 both recognize ssRNA as well as imidazoquinoline compounds like imiquimod and resiquimod that act as potent synthetic agonists that boost anticancer as well as antiviral responses, especially in mammals (147). Most higher vertebrates possess single gene orthologs of TLR7 and TLR8, a case that is not consistent across most fish species which often possess two or more isoforms of TLRs 7 and 8 including common carp with three duplicates of TLR8 and two TLR7 copies, Atlantic cod with three TLR7 copies and a shocking twelve TLR8 copies, and channel catfish with two TLR8 copies as depicted in **Figure 2A** (88, 99). Mammalian TLR7 and TLR8 possess around twenty-seven LRRs, whereas fish have significantly fewer, such as in large yellow croaker, which possesses seventeen TLR7 LRR motifs and fourteen TLR8 LRR motifs, and cyprinid TLR7 and TLR8 containing fifteen and seventeen LRR motifs (80, 93, 148). TLR7 and TLR8 can form homodimers as well as the TLR7/8 heterodimer to recognize a wider range of nucleic acid PAMPs. The mammalian TLR7/7 homodimer has been modeled using computational methods, and simulations have predicted that resiquimod is its most effective agonist, sharing structural similarities with the TLR8/8 homodimer (149). Similarly, resiquimod is an agonist of the fish TLR7/8 heterodimer that has been shown to inhibit viral infection by megalocytivirus through its activation of the TLR7/8 receptor when used as an immunostimulant in Japanese flounder (150). A novel function of TLR7 was recently identified as the fish-specific ability to sense not only ssRNA, but also dsRNA in lysosomes, though the exact mechanisms remain unexplored (151). Beyond its importance for viral response, TLR7 in tongue sole is highly involved in antibacterial immunity as it is upregulated by bacterial infection and downregulated when infected with a viral pathogen, demonstrating the broadened diversity of ligand-binding functionality across teleost TLRs (152). Beyond these examples, TLR7 is also found in olive flounder (*Paralichthys olivaceus*), common carp, gilthead sea bream (*Sparus aurata*), rainbow trout, grass carp, and other aquaculture species (152). TLR8 tends to somewhat follow these paths of conservation between fish and mammals, with more duplication events as compared to teleost TLR7, reflecting functional specialization; regardless, these members of TLR family 7 remain under strong positive selection with few instances of gene loss due to their important role in nucleic acid recognition across vertebrate species (153).

TLR9 is another endogenous member of the TLR family 7 whose role in sensing unmethylated CpG DNA motifs from varying pathogen sources remains conserved between higher and lower vertebrates. Short, synthetic single-stranded DNA molecules that mimic microbial DNA, called CpG Oligodeoxynucleotides (ODNs), are also agonists of TLR9 and can be designed to bind based on lineage-specific ligand preferences. This is an important fact when considering the diversity across fish TLR9 isoforms, which often preferentially bind distinct CpG motifs from their mammalian homologues. TLR21, which is absent in mammals but present in avian and fish species, also binds CpG motifs, although the sequence specificity is often different from TLR9 (154). Binding occurs in the proteolytically cleaved ectodomain of the TLR9 homodimer upon sensing of not only CpG motifs but simultaneously has been observed to recognize the 5’ -xCx DNA motif that forms a stable 2:2:2 complex in mammals (155). Although yet to be confirmed in fish, this finding could be tested and potentially inform the development of immunomodulators of TLR9 in fish. TLR9 functions exclusively as a homodimer in both fish and higher vertebrates, with high rates of conservation in their extracellular LRR domain, which range between 12–17 LRR motifs in various bony fish species and 18 LRR motifs in human TLR9 (156, 157). Although TLR9 functions primarily as a positive regulator of immune signaling in mammals, due to the duplication events that have occurred within the TLR family 7, alternative duplicate isoforms of TLR9 in orange spotted grouper have revealed that TLR9b functions as a molecular sink by competing with TLR9a for ligands despite having a non-functional TIR domain (158). This mechanism is considered to have been acquired recently in fish, suggesting this diversification in regulatory signaling to be potentially present in other species with similar TLR9 conservation patterns (158). TLR9 is a useful target for adjuvant development and has been extensively evaluated across higher and lower vertebrates for its potent immunostimulatory qualities. As elaborated on in later sections, the role of this receptor is expanded in fish species due to its further cooperation with TLR21. TLR9 is absent in the earlier mentioned bird and lamprey species but present and functional in most vertebrate genomes, such as Atlantic salmon, sea bream, turbot, zebrafish, common carp, rainbow trout, and channel catfish (seen in **Figure 2A**) (154, 159). The proposed signaling pathway of teleost TLR9 is further detailed in Section 5.2.1 in response to GACGTT/AACGTT motifs, which bind TLR9 preferentially, signaling through the MyD88-dependent pathway to trigger B-cell activation and proliferation (160–162).

**Figure 3:**
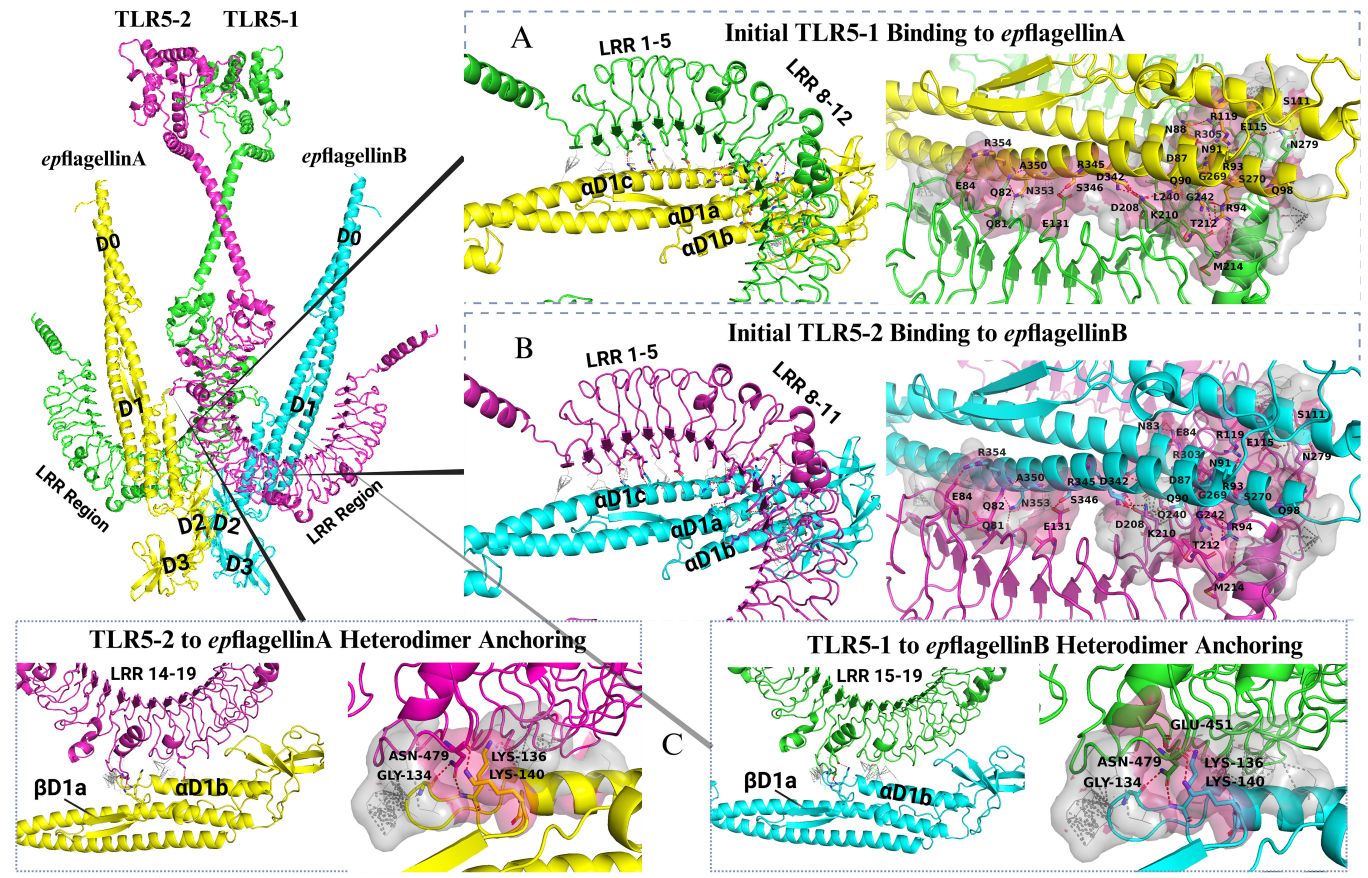
Structural model of *Edwardsiella piscicida* flagellin (*ep*flagellin) binding to TLR5–1 and TLR5–2 heterodimer of Channel catfish (*Ictalurus punctatus*). The modeled complex depicts the heterodimeric association between two *E. piscicida* flagellin monomers, epflagellinA (yellow) and epflagellinB (cyan), and two distinct membrane-bound TLR5 homologs, TLR5-1 (green) and TLR5-2 (purple). EpflagellinA and epflagellinB represent identical flagellin subunits individually labeled to describe their respective interaction sites within the receptor complex. Each flagellin monomer associates with one TLR5 molecule, yielding a cooperative epflagellin–TLR5–1 and epflagellin–TLR5–2 pairing that together stabilizes the overall heterodimeric assembly. Panels A and B highlight the molecular interfaces corresponding to the initial docking, while panel C displays the subsequent anchoring of each epflagellin to stabilize dimerization. Left subpanels illustrate the broader contact regions between the D1 elements of epflagellin and the concave LRR surfaces. Right subpanels display TLR residue interactions, where hydrogen bonds are represented by red dashed lines with red shaded contours and van der Waals interactions by grey dashed lines with grey shading. Labeled residues are representative of the TLR. Protein sequences for TLR5-1, TLR5-2, and epflagellin were retrieved from NCBI under accession numbers AEI59668.1, AEI59669.1, and WLJ42332.1 and submitted together as FASTA sequences to AlphaFold Server v2.3 for simultaneous multimer prediction of the complete 4-subunit complex. The prediction yielded an ipTM of 0.63 and pTM of 0.65, with per-residue pLDDT scores predominantly >90 in the LRR binding regions. The predicted interface was consistent with the zebrafish TLR5–flagellin complex (PDB: 3V47) (56). Structures were imported into PyMOL v3.0 without energy minimization for visualization and figure preparation. Interface contacts were classified using LigPlot+ v2.2 (253). LRR boundaries were identified using LRRsearch v1.0, all other domains using SMART v10 (72, 74). The figure was created in BioRender.com (72). All interactions shown are computationally predicted and require experimental validation.

#### 3.2.6 TLR family 11

The TLR family 11 includes TLRs11-13, TLR16, TLR19, TLR20, TLR21, TLR22, TLR23, and TLR26. There are limited representatives in higher vertebrates, aside from TLR11 and 12 in mammals and birds, TLR13 in mice and rats, and TLR21 in birds. TLR family 11 represents the most expanded family in teleost species, with the highest rates of individual gene duplication and numerous instances of fish-specific expansions. Although these novel proteins are not as well characterized as the typical mammalian homologues, understanding the mechanisms behind how fish have adapted these diverse lineage-specific functions can provide novel therapeutic targets for research. We will not cover TLR11 and TLR12 due to their absence across fish species and the lack of direct orthologs, but rather focus on the fish-specific TLRs present in TLR family 11 (8).

TLR19 is fish-specific, localized intracellularly in the endosomal membrane, and synthesized in the ribosomes rather than following the typical endoplasmic reticulum trafficking (163). TLR19 also colocalizes with TRIF to sense dsRNA, triggering antiviral immune responses by increasing IFN-1 activity upon sensing of agonists like poly I:C in common carp (7). A recent study, however, has suggested that barbel chub isoforms of TLR19 could signal through the IRF3-MyD88 pathways upon infection with GCRV, suggesting the existence of lineage-specific variations in signaling mechanisms between teleost (164). Beyond its role in the antiviral response, TLR19 has also been observed to upregulate upon stimulation with *A. hydrophila* species in yellow catfish as well as *Edwardsiella ictaluri* in channel catfish, implicating it in antibacterial immunity as well (7). The number of LRRs varies significantly between fish species, with twenty-six LRR motifs in common carp and Atlantic salmon TLR19, zebrafish possess twenty-four LRR motifs, and grass carp and barbel chub (*Squaliobarbus curriculus*) contain nine and ten LRR motifs, respectively (7). The high variability of TLR19 has enabled species-specific innate defenses for the dynamic aquatic environments of teleost fish. TLR19 preferentially clusters with TLR20, its closest paralogue, and TLR11/12 on an evolutionary tree (165).

TLR20 is a fish-specific member of TLR family 11, often found co-localized with TLR19, which is absent in higher vertebrates, that are involved in broad pathogen recognition, though its specific ligand remains unknown. The subcellular localization of TLR20 is intracellular, where it responds to a variety of viral, bacterial, and parasitic infections through various adaptor protein-mediated pathways depending on the specific TLR20 isoform (166). For example, common carp possess three TLR20 proteins (TLR20.1, TLR20.2, and TLR20.3) that are distinct from one another, exhibiting varying numbers of LRR motifs that potentially affect whether they are mediated by MyD88 or TRIF, as well as possessing a truncated TLR20.1 isoform that lacks most of its extracellular domain (71). This truncation also occurs in channel catfish, which possess two TLR20 genes, TLR20–1 and TLR20-2. Catfish TLR20–2 lacks a significant portion of its extracellular domain and has been hypothesized to lack functional capacity, acting as more of a pseudogene (8). By examining the computational model illustrated in **Figure 2A** and comparing it with the shared features of truncated grass carp TLR20.1, we can corroborate the theory that TLR20–2 is most-likely non-functional in channel catfish; although experimental validation is still needed to confirm (167). Beyond these duplications, copies of TLR20 are common across fish species, with examples such as Atlantic salmon, which possess four copies (TLR20a-d) and zebrafish, which possesses four functional TLR20 copies (TLR20a-d) and two pseudogenes (168). The specific structure of TLR20 members across fish lineages is also weakly conserved, with distinct LRRNT cysteine motifs and variations in binding domains that may possess between twenty-six and twenty-eight LRRs in functional orthologs, suggesting potential functional variations between isoforms (168). TLR20 is reported ubiquitously across various teleost species, including zebrafish, Rainbow trout, Atlantic salmon, channel catfish, common carp, and others (71).

TLR21 is a non-mammalian TLR9 functional homolog found exclusively in amphibian, avian, and fish species that is conserved structurally across fish species to recognize unmethylated CpG DNA motifs and CpG ODN agonists to activate NF-κB and IFN production (169). The localization of TLR21 is conserved with TLR9 as it is intracellularly expressed in endosomal compartments, requiring the same chaperone proteins for trafficking to endosomes (154). TLR21 was recently observed to regulate immunity via the TRIF-dependent pathway in Largemouth bass, bypassing the MyD88 pathway altogether, a mechanism we interpret further in Section 5.2.1 (170). This finding remains to be confirmed in other teleost species but would reveal key insights into the role of TLR21 in fish pathogen response. Beyond fish TLR21’s ability to bind CpG motifs, the functional pathogen response of TLR21 has expanded for broad antiviral and antibacterial recognition of PAMPs like LPS and poly I:C through distinct LRR binding sites (171). TLR21 has also been observed to upregulate upon parasitic infection of protozoan ectoparasite *Cryptocaryon irritans*, implicating its importance for response against a wide range of aquatic pathogens (8). The extracellular region of TLR21 is conserved across fish, with Japanese pufferfish, zebrafish, and channel catfish all possessing 23 LRR motifs, whereas avian species like chicken possess twenty-seven LRRs (172, 173). All birds and most fish have a single copy of the TLR21 gene with lineage-specific exceptions in the orange spotted grouper, which possesses two copies, TLR21A and TLR21B, as well as the Antarctic cod fish *Gymnodraco acuticeps* having TLR21–1 and TLR21-2 (174, 175). TLR20 has a widespread presence across teleost species, including zebrafish, rohu, orange-spotted grouper, yellowtail, large yellow croaker, Grass carp, channel catfish, common carp, and more (8, 171).

TLR22 is a fish/amphibian-specific receptor found predominantly intracellularly in the endosomal and lysosomal membrane of immune cells; although it has been reported that Japanese pufferfish TLR22 localizes to the cell membrane surface (176). TLR22 is most often considered for its protection against ectoparasite infections, as well as being able to recognize various viral, poly I:C, and bacterial infections in numerous fish species (50, 177). TLR22 primarily signals through the MyD88 pathways to upregulate pro-inflammatory cytokine and IFN production (176). However, studies in orange-spotted grouper have also identified its important role as a regulator and equalizer of the antiviral immune responses through its upregulation of the extracellular signal-related kinases (ERK) pathways, negative regulation of the c-jun amino-terminal kinases (JNK), and p38 MAP kinases. Additionally, the NF-κB pathway of TLR22 has appeared to be affected by the length of poly I:C (178). This immune regulation allows TLR22 to control the excessive generation of reactive oxygen species, avoiding over-inflammation that could result in host damage (179). The number of LRRs varies between species, with common carp possessing sixteen LRR motifs, channel catfish possessing fourteen LRR motifs, and zebrafish, Japanese pufferfish, and grass carp possessing fifteen, as well as olive flounder possessing nineteen LRR motifs, suggesting lineage-specific functionalities (50). This is further supported by the frequent duplication of TLR22 across fish lineages such as two TLR22 copies in rainbow trout (TLR22 and TLR22L), three in common carp (TLR22-1, TLR22-2, and TLR22-3), and in Atlantic cod which possess twelve unique duplicates of TLR22 (TLR22a-l) that likely arose through tandem duplication events (99, 180, 181). Zebrafish TLR22 is also uniquely elastic in its ability to twist at either terminal, allowing it to interact with diverse dsRNA viruses through its binding sites at LRRNT-LRR3 and LRR 22–24 in TLR22 (121).

Another member of the fish-specific expanded TLR family 11 is TLR23. TLR23 is intracellularly located and able to signal through interactions with both MyD88 and TRIF, promoting the upregulation of inflammatory cytokines, IFNs, and antimicrobial peptides. Nile tilapia TLR23 has been shown to respond to a variety of viral and bacterial agonists, including poly I:C, *A. hydrophila*, and LPS inducing the expression of IL-12a, TNFα, IFNd2.13, and IFNd2.8 (182). TLR23 is duplicated in mudskipper (*Periophthalmus barbarus*) with 7 copies of TLR23 (TLR23a-g) as well as in Atlantic cod with two distinct paralogs, TLR23a and TLR23b, that follow the trend of Atlantic cod expansion of TLR family 11 (180, 183). Sundaram et al. (2012) speculates that the TLR23a paralogue may be the ancestral protein, and the TLR23b that possesses fewer LRR motifs acts as a redundant copy with significantly lower expression patterns as compared to those of TLR23a. TLR23 orthologs cluster with TLR22, reflecting a close phylogenetic relationship despite distinctions in their functionality (183). The length of TLR23 orthologs varies greatly due to their expansion between fish lineages, with ten to sixteen LRR in mudskipper TLR23s as well as twenty-seven and fourteen LRR motifs in Atlantic cod TLR23a and TLR23b (180, 183). TLR23 is very well conserved across teleost species, with the exception of channel catfish, which lacks a TLR23 gene altogether (8). TLR23 has been identified across most evaluated fish species, including Atlantic cod, Japanese pufferfish, Mandarin fish, mudskipper, and many others (182).

TLR26 is a recently discovered fish-specific TLR whose precise ligand-specificity has yet to be identified. It was originally identified as a singular copy in channel catfish but is also expressed in yellow catfish. In both fish species, TLR26 is most closely related to TLR20, another fish-specific TLR that senses a wide variety of microbial PAMPs (17). Currently, the only evaluation of TLR26 immune regulation has been done in yellow catfish where it was upregulated at various levels in different immune tissues upon response to poly I:C and LPS challenge, which could implicate it as a potential functional paralog of TLR20, but studies in other teleost species and with other ligands are necessary to determine the exact functionality of TLR26 in fish (184). There are also varying reports on the exact structure of TLR26 extracellular domain, with Quiniou et al. (2013) reporting twenty-nine LRR motifs in channel catfish, whereas our evaluation using LRRsearch.com to visualize channel catfish TLR26 in **Figure 2A** revealed fewer at seventeen LRR motifs which may be a result of variations in definition of LRR residue across different models (8, 184).

The discovery and classification of fish-specific TLRs, including TLRs18–20 and TLRs22-28, was instrumental for discerning the unique expansion of fish innate immunity and clarified why adaptive immunity plays a less central role in their pathogen responses than in higher vertebrates.

### 3.3 Evolutionary insights into TLR functionality

TLRs constitute an ancient, evolutionarily highly conserved family of pattern-recognition receptors found across both invertebrates and vertebrate species. Cross-phylogenetic analysis has revealed an estimate of more than 600 million years of TLR evolution originating prior to the divergence of bilaterians and cnidarians, emerging from a common ancestor of all “true” animals potentially more than 700 million years ago (185).

As extensively discussed earlier, the number, functionality, and structure of chordate sccTLRs have evolved to vary greatly between higher and lower vertebrates, likely in response to the free-flowing, diverse pathogen-rich environments that fish inhabit as compared to mammals. The earliest components of the innate immunity arose around 1 billion years ago, prior to the evolution of the adaptive immune system that occurred only 450 million years ago in the ancestor of chordates, expanding immunity to include the development of antigen-specific memory (185, 186). **Figure 2B** illustrates these conservation patterns across vertebrates as several members of each TLR family are shared across various reptile, fish, bird, and mammal species with isoform-specific exceptions such as TLR21 only being present in chicken, frog, and channel catfish (**Figure 2A**) whereas TLR2, TLR3, and TLR5 isoforms are found across all key groups. Although teleost fishes contain a complete adaptive immune system possessing key components like B and T lymphocytes, immunoglobulins, and MHC molecules capable of providing long-term memory defenses against pathogens, their cell-mediated response times can be shorter-lived and not as potent as those of higher vertebrates like mammals, birds, amphibians, and reptiles (187). Factors such as extreme temperature and chemical environmental conditions, as well as constant exposure to large pathogen loads, have likely driven the reliance of fish on their robust innate defenses. These environmental stressors and evolutionary pressures have driven lineage-specific TLR sequence modifications, TLR gene duplications, the diversification of antimicrobial peptides, a more active alternative pathway complement system, and specialized immune organs such as head kidneys (175, 188–190).

The number of TLR families (six) has remained consistent between higher and lower vertebrates, with organisms like fish that contain extensive duplicate isoforms classified within their respective gene families based on phylogenetic analysis (8). The coevolution of these host-pathogen responses is often considered the driving force behind the purifying selection of conserved TLR genes and features like the TLR2 heterodimer formation, as well as the elimination of redundant copies like the pseudogenes found in those like TLR20.2 in channel catfish (191).

To cover a few key examples of evolutionary events driving TLR evolution, we can begin with a strong example in TLR subfamily 1, which contains the highest number of members across all vertebrates: TLR1, TLRs1A/1B, and TLRs2A/2B in birds, TLR2, TLRs6, and 10 in mammals, and TLRs14, 18, and 24–28 in fish (26). This diversification and expansion of TLR family 1, especially in novel fish TLR genes, reflects the class-specific adaptations that have evolved in teleost innate immune defenses. The formation of heterodimers is another conserved feature of TLR family 1 receptors, with fish TLR1 and TLR2 likely cooperating due to comparable patterns of upregulation from bacterial infection as well as similar expression patterns dispersed across immune-related tissues (83, 192, 193). This hypothesis, however, requires further experimental confirmation, which is an excellent opportunity for the application of ligand binding investigations using *in silico* techniques to model predicted crystal structures, such as those done in Section 5 in TLR5, TLR9, and TLR21. To speculate on the absence of TLR 6 and 10 in teleost fishes, studies have implicated that the tandem duplication event(s) that created TLRs 6 and 10 in mammals may have taken place after a splitting off from the teleost and lobe finned fishes lines around 400–450 MYA prior to the appearance of the tetrapod lineage from a common ancestor through phylogenetic testing (78).

Beyond the TLR family 1, the subfamilies containing TLR 3, TLR4, TLR5, and TLR7 are shared by all vertebrates. TLR3 of the TLR3 family is thought to be the most ancient, with the highest likelihood of purifying selection, seeing as it is the only member of its family functioning as a homodimer and has been found in every vertebrate genome. It has also been suggested that the TLR3 clade could have originated before even the evolution of deuterostomes when observing trends using comprehensive comparative relationships on phylogenetic trees (26, 52).

The TLR family 4 is similarly well conserved in teleost, likely originating before jawed vertebrates with evolutionary variation due to instances of complete gene losses, such as in Japanese pufferfish, and instances of gene duplications like the TLR4-1 and TLR4-2 (also known as TLR4s) isoforms in channel catfish, as seen in **Figure 2A** illustrating how the lack of TM and TIR domains of TLR4-2 (TLR4s) has adapted this TLR4 copy to function as a putative soluble form (8, 92). Host-pathogen coevolution has likely driven the appearance of fish-specific soluble TLR isoforms, which contrast in functionality and sequence from human soluble isoforms like human sTLR2 and sTLR4 (194, 195).

The overall family of TLR5 is well conserved in fish, with few instances of complete gene loss besides Atlantic cod. However, there have been notable instances of functional variations and gene duplications within specific teleost lineages. Some TLR5-like genes in fish act as nonfunctional pseudogenes, such as those in clownfish and gene duplications in the zebrafish genome (TLR5a and TLR5b, which share much of their identity), as well as three forms in the channel catfish genome, as seen in **Figure 2A** (TLR5-1, TLR5-2, and soluble TLR5s) (26). Contrastingly, most higher vertebrates contain a single functional copy (144).

Novel fish-specific TLRs continue to be actively identified, and phylogenetic analysis of sequence composition and function between species has allowed for the organization and classification of variations that inform the development of more tailored immunotherapeutics in aquaculture (17). By investigating the evolutionary histories of these ancient genes, we can draw more informed comparisons to their more well-understood mammalian counterparts.

## 4 Known TLR agonists

The TLR profiles, signaling pathways, and structural features described in preceding sections provide a basis for evaluating known TLR agonists as adjuvant candidates. The following agonists are reviewed here not only for their immunostimulatory properties, but as a translational guide for adjuvant selection based on receptor specificity, species compatibility, and practical vaccine delivery considerations in aquaculture.

### 4.1 Polyinosinic: polycytidylic acid

Poly I:C is a synthetic double-stranded RNA analogue molecule that mimics viral RNA and is recognized predominantly by TLR3, although it is able to elicit other TLRs as well as MDA5 (Melanoma Differentiation-Associated protein 5) and Retinoic acid-inducible gene I/(RIG-I)-like receptor 1 (RIG-1) (196, 197). Through its association with TLR3, poly I:C can activate the TRIF-dependent as well as MyD88-dependent signaling pathways, leading to the activation of pro-inflammatory cytokines and induction of antiviral IFNs, respectively (53). Poly I:C has proven useful across various higher and lower vertebrate species for its formulation alongside viral vaccines since it structurally resembles the double-stranded RNA helices found in many viruses. Several studies have also suggested its potential as an immunostimulant for enhancing mucosal immunity as it produces antigen-specific antibodies within mucosal tissues (198). Although they are not currently formulated with commercially available vaccines for humans or fish, poly I:C is available for use in pre-clinical and clinical trial settings as immunostimulants against a variety of cancer and human infectious diseases (199). Within exclusively teleost studies, its already potent immunostimulatory effects are further enhanced by encapsulation into nanoparticles and the creation of fish oil emulsions for oral vaccine development, optimizing aquaculture vaccine delivery systems (198, 200). Experimental evaluation and immunomodulation using poly I:C has been done in many fish, including Japanese Flounder, rohu, grass carp, zebrafish, and several others (200–203).

### 4.2 CpG ODNs

CpG Oligodeoxynucleotides (CpG ODNs) are synthetic DNA molecules that mimic the unmethylated CpG motifs that are often found in viral and bacterial genomes (204). In fish, TLR9 and TLR21 are both able to recognize and bind CpG ODNs with differences in expression patterns depending on species-specificities and sequence recognition profiles. For example, TLR9 in fish preferentially binds CpG GACGTT and AACGTT motifs, which more closely resemble the mouse TLR9, while fish TLR21 prefers GTCGTT motifs, aligning more with human TLR9 (154). These findings are included in the proposed comparative signaling pathway in Section 5.2.1 and the homodimer structures of channel catfish TLR9 and TLR21 in Section 5.2. CpG ODNs signal mostly through the MyD88 pathway, although there is discourse surrounding TLR21’s potential signaling through the TRIF-dependent pathway, as well as inducing the maturation of antigen-presenting cells (APCs) (205). These examples showcase the diverse modes of CpG motif recognition between higher and lower vertebrates and reflect the expanded repertoire of fish TLRs that evolved to detect a broader range of PAMPs, like the distinct CpG motifs, which is necessitated by the pressures of their unique aquatic environments. CpG ODNs in mammals as well as fish promote a Th-1-based immune response (206). CpG motifs upregulate MyD88 and its pro-inflammatory pathways primarily, inducing the expression of Mx antiviral proteins, chemokine CC, and interleukin IL-1*β* specifically in fish evaluations (207). They have been approved for commercial use as an adjuvant in human vaccine formulations specifically for HepB (heplisav-B) vaccines within the United States as of 2024 (208). However, CpG ODNs are not yet commercially used in aquaculture; nonetheless, they are widely researched, showing promise as standalone prophylactics as well as in conjunction with inactivated and DNA vaccines for fish-specific infections (209). It is worth mentioning that there are different reactivities and sequence specificities between species, requiring further research and trial testing. As it stands, there is no universal CpG motif for all teleost species, and the optimal sequence for immune stimulation varies between species, presenting another challenge for adjuvant development (154, 210, 211). They have been immunologically evaluated across many fish species, including rainbow trout, Atlantic Salmon, orange-spotted grouper, zebrafish, cobia (Rachycentron canadum), and many more (154, 158, 162, 207, 212).

### 4.3 Flagellin

Flagellin is a structural protein component of the bacterial flagellum that enables the propeller-like motion facilitating bacterial movement toward more favorable conditions and away from toxic compounds. It is recognized in mammals as well as fish on the cell surface of monocytes, macrophages, and epithelial cells by binding favorably to TLR5 through the MyD88-dependent pathway (213). Beyond the conserved TLR5 homodimer recognition of flagellin, NLR Family CARD domain-containing protein 4 (NLRC4) can bind flagellin through the phosphorylation of Ser533 and activation of NAIP5, forming the inflammasome in higher and lower vertebrates to elicit pro-inflammatory cytokines IL-1β and IL-18 (214). Certain fish species also have duplicate TLR receptors that allow for different modes of regulation and immune stimulation, such as the heterodimerization of membrane TLR5a and TLR5b in zebrafish, and TLR5–1 and TLR5–2 in grass carp and the suppression of overactivation by soluble TLR5s that block further inflammatory signaling (215). A model of this heterodimeric structure of channel catfish is further detailed in Section 5.1. Flagellin recognition by TLR5 isoforms induces a potent inflammatory response by the expression of TNF-α, IL-1B, IL-6, IL-8, IL-11, M17, IL-17C, IL-12, IL-22, and IL-23 (137, 216). For this reason and the localization of TLR5 in epithelial cells, flagellin has high immunostimulant efficacy via non-invasive mucosal routes, making it a valuable tool for creating practical vaccines for aquaculture and veterinary use (217, 218). Flagellin has entered human clinical trials for its stability and use within mixed antigen formulations as well as built-in protein antigen fusions, but is currently not commercially available for either human or aquatic medicine as a result of dosing challenges leading to high reactivity at increased concentrations (219). The immunogenicity of flagellin has been extensively studied across various fish species, for instance, in salmon, zebrafish, large yellow croaker, Indian major carp, and so on (132, 216, 220, 221).

### 4.4 LTA

Lipoteichoic acid (LTA) is a cell wall component of gram-positive bacteria that varies somewhat between different species but is composed of a glycolipid anchor linked to a repeating alditol polymeric backbone (222, 223). LTA and its derivatives are agonists of TLR2, more specifically the TLR2/1 heterodimer found on the surface of immune cells, where it triggers a signaling cascade of various cytokines and immune mediators, including TNF-α, IL-1β, and inducible nitric oxide synthase (iNOS) in mammals and fish (224). It has also been hypothesized that the TLR2/14 fish-specific heterodimer could be capable of detecting LTA, but this has yet to be experimentally confirmed (225). Several LTA strains have been tested as immunostimulants in fish and vary somewhat in their ability to induce robust immune responses (226). LTA adjuvant derivatives have used the conserved structure of their poly-1,3-(glycerophosphate) backbones to formulate vaccines against a wide range of nosocomial bacterial infections like *Enterococcus faecium* and *Clostridium difficile* (227, 228). They are not currently FDA-approved for formulation within commercial vaccines for human or veterinary medicine. However, advancements in the use of LTA derivative adjuvants are limited by the need to address concerns surrounding the structure variability and potential for toxicity (228). LTA has been evaluated and confirmed as an immunostimulant in fish species, such as common carp, Indian major Carp, turbot, mandarin fish, Dabry’s sturgeon, etc. (225, 229, 230).

### 4.5 Pam3CSK4

Palmitoyl-3-Cysteyl-4-Lysine (Pam3CSK4), also known as Pam3, is a synthetic triacylated lipopeptide characterized by its three fatty acid chains that mimics the N-terminal tripalmitoylated cysteine of bacterial lipoproteins (231, 232). Pam3 is bound by predominantly the TLR1/2 heterodimer in mammals as well as fish, activating the NF-κB and MAPK pathways– inducing the expression of pro-inflammatory cytokines IL-6 and TNF-α and chemokines monocyte chemoattractant protein 1 (MCP1) and c-x-c motif chemokine ligand 1/8 (CXCL1/8) (233). Mammals are able to additionally bind Pam3 through TLR2/6 heterodimer activation. Pam3 promotes primarily a Th-2-based immune response for a strong humoral response by B cell stimulation, which has been exploited in a variety of cancer and infectious disease vaccines in human immunological research and clinical trials (206, 234). Fish-specific studies most often used Pam3 as an adjuvant to boost the immune response to bacterial infection but show mixed to low results in the level of inflammatory response and are thus not used in fish veterinary research as often as in human medicine. Neither is approved as of today for formulation for human medicine or veterinary industry applications (235). For example, Pam3 shows potential as an adjuvant for live-attenuated viral vaccines for the human Influenza virus, but has shown somewhat lower levels of cytokine expression in fish reports (231, 236).

Lipopeptides are abundant in fish pathogens, making them good cross-species agonists and have been explored and implicated for mixed-adjuvant vaccine formulations that would provide more robust, long-lasting immune responses (204). Although they have been investigated to a lesser extent in fish, Pam3 has nevertheless been evaluated in rainbow trout, common carp, and zebrafish (53, 235, 237).

The closely related analog, Palmitoyl-2-Cysteyl-4-Lysine (Pam2CSK4), also known as Pam2, is another synthetic lipopeptide mimicking the amino terminus of diacylated gram-positive bacterial lipoproteins and signals through the TLR2/6 heterodimer in mammals, and through TLR2/1 and potentially TLR2/14 in fish, reflecting the expanded TLR repertoire of teleosts (232). Like Pam3, Pam2 activates NF-κB upon heterodimer sensing, driving production of inflammatory cytokines such as TNF-α in murine models, though it has yet to be evaluated in fish studies (238). Pam2 promotes primarily Th2-based immune responses, making it useful for enhancing immunogenicity against extracellular pathogens like parasites (206). While Pam2 has entered preclinical and clinical trial settings with little to no reported side effects, it is generally considered weakly immunogenic relative to Pam3 and is not currently used in any commercial human or fish vaccines; as its application in fish vaccine research remains largely unexplored, it is discussed here as a subordinate analog to Pam3 rather than warranting its own section (239).

### 4.6 Imiquimod and resiquimod

Imiquimod and resiquimod are classes of imidazoquinolines, which are synthetic compounds that stimulate the immune system, often used as topical therapeutics as well as superficial cancer research in mammals and used exclusively as immunostimulant agonists in fish where they enhance antibacterial and antiviral responses through the induction of TLR7 homodimers and TLR7/8 heterodimers (150, 240). Within human medicine, imiquimod and resiquimod are commonly found in topical ointments as immune response-modifying treatments for actinic keratosis, pre-cancerous therapeutics with resiquimod verified as the more potent immunostimulant molecule (241). Imiquimod, through the MyD88 pathway, produces cytokines such as IFN-β. Resiquimod similarly signals through the MyD88 pathway while producing cytokines and chemokines, including IFN-γ, TNF-α, IL-10, chemokine (C-C motif) ligand 4 (CCL4), and B lymphocyte stimulator (BlyS) (124, 150). These imidazoquinolines activate and enhance T cell response through a Th-1-based approach that matures dendritic cells, encouraging a robust, durable immunity (242). Certain mammalian trials for their use alongside DNA vaccination have enhanced the potency and longevity of immune system responses, illustrating the successes of targeting TLRs in the adjuvant development process (242). Neither imiquimod nor resiquimod is commercially used as a vaccine adjuvant in fish or mammals, besides their FDA approval for their other medical uses in human medicine, but have been determined to be potent immunostimulants for fish immunomodulation in a variety of species, including channel catfish, Atlantic halibut, Japanese flounder, and golden pompano (124, 150, 243, 244).

### 4.7 LPS and MPLA

Lipopolysaccharide (LPS) is a major outer membrane component of Gram-negative bacteria composed of lipid A, a core oligosaccharide, and an O-antigen polysaccharide (245, 246). Many important fish pathogens, including *Aeromonas*, *Edwardsiella*, and *Vibrio species*, contain LPS, making it relevant to aquaculture immunology and vaccine development (246, 247). LPS has been widely used in fish as an experimental immunostimulant capable of inducing inflammatory cytokine expression, leukocyte activation, macrophage responses, and enhanced antibacterial immunity (248). Unlike more receptor-specific agonists, however, the immunostimulatory effects of LPS in teleosts are not consistently linked to a conserved mammalian-like TLR4 pathway, and responsiveness varies substantially between fish species (249). As a result, LPS is more appropriately viewed as a broad innate immunostimulant in fish rather than a universally conserved TLR4 agonist (249).

Monophosphoryl lipid A (MPLA) is a detoxified lipid A derivative that retains much of the immunostimulatory activity of native LPS while substantially reducing endotoxin-associated toxicity (250, 251). Because of its improved safety profile, MPLA represents a more practical framework for lipid A-based adjuvant development in aquaculture vaccines. However, its mechanism in fish should still be interpreted cautiously due to the species-specific nature of teleost LPS sensing pathways (252).

## 5 Molecular docking and interaction analysis

Teleost TLRs recognize specific pathogenic elements, such as various forms of TLR5 acting as flagellin receptors, as well as TLR9 and the fish-specific TLR21 acting as the CpG DNA sensing receptors. In channel catfish, these receptors are known to participate in innate immune activation, yet the structural basis underlying ligand engagement, receptor pairing, and stabilization remains incompletely defined. To address this gap, *in silico* models were performed via molecular docking and interaction analyses to model *Edwardsiella piscicida* flagellin recognition by TLR5 paralogs, TLR5–1 and TLR5-2. Along with this, binding of synthetic CpG DNA by TLR9 and TLR21 provides comparative insights into receptor-specific ligand interfaces and dimerization mechanisms. These computational analyses function as a virtual screening framework for adjuvant optimization, enabling prioritization of ligand candidates based on predicted binding affinity and receptor specificity before committing to costly *in vivo* validation. A signaling pathway was further employed to demonstrate TLR9 and TLR21 responses to CpG DNA motifs.

### 5.1 Structural basis of *ep*flagellin recognition by TLR5–1 and TLR5-2

The *E. piscicida* flagellin (*ep*flagellin) represented in **Figure 3** follows a conserved multi-domain organization observed in bacterial flagellins. It consists of four domains, which include the D0 and D1 domains and the peripheral D2 and D3 regions. The D0 and D1 domains form a central helical core that polymerizes into the flagellar filament and initiates host immune recognition via TLR5 (55, 253, 254). In teleost fish, TLR5 recognition is better described as detection of conserved structural features in flagellin’s D1 region, not an identical amino acid sequence shared by all bacterial flagellins (55, 253). Sequence changes in conserved flagellin regions can weaken or evade TLR5 activation, as shown in TLR5-evasive flagellins such as *Helicobacter pylori* flagellin (255, 256). Many bacteria also glycosylate flagellin, but these modifications are commonly located in the variable, surface-exposed D2/D3 regions rather than the main TLR5-recognition interface, so glycosylation does not generally abolish TLR5 recognition (257). However, this should be stated cautiously for fish because direct teleost-specific glycosylation studies are limited, and in some bacterial contexts, glycosylation can reduce or alter TLR5 activation depending on how it affects flagellin structure or receptor accessibility (258).

In contrast, the D2 and D3 domains, which are present in the *ep*flagellin sequence, tend to extend outward from the helical axis and largely consist of antiparallel β-sheet structures (55, 253). These domains exhibit high sequence variability and antigenic diversity among bacterial species (192, 254, 259). Because they do not participate in TLR5 recognition or heterodimerization, their detailed structural characterization lies beyond the scope of this analysis.

Among fish TLRs, TLR5 presents a particularly interesting case because of its duplication into membrane-bound TLR5-1, TLR5-2, and the fish-specific soluble form, TLR5S, which shares a high degree of sequence homology (192). Structural docking studies, such as the model shown here, generate testable hypotheses about how these paralogs may cooperate in flagellin recognition, while building on the limited experimental evidence currently available in channel catfish. Although wet-lab data confirm that flagellin engagement of TLR5 activates NF-κB and JAK-STAT pathways, they do not establish whether TLR5–1 and TLR5–2 function as homodimers or as obligate heterodimers (254). Insights from zebrafish, where duplicated TLR5 isoforms heterodimerize to form the active 2:2 receptor–flagellin complex, suggest a similar mechanism may occur in catfish, but this remains to be validated *in vivo* (253, 259). Notably, soluble TLR5 (TLR5-S) docking is not shown in **Figure 3**; however, the protein is hypothesized to act by binding extracellular flagellin and enhancing presentation to the membrane receptors (TLR5–1 or TLR5-2), potentially forming a heterodimer and thereby increasing sensitivity, though this remains experimentally unconfirmed in catfish (260).

Regardless of isoform usage, the predicted binding process displayed in **Figure 3** suggests bonding of flagellin’s D1 domain to the concave face of TLR5’s LRR domain, followed by stabilization through the D0 domain. Also, as mentioned, the TM domain is responsible for heterotypic interactions between individual TLRs upon binding (66). Both of these processes are highly conserved across vertebrates, supporting the use of comparative models for studying fish-specific adaptations (66, 254, 259). In the case of channel catfish TLR5 homologs interacting with *ep*flagellin, the binding process involves the initial interactions of flagellin’s D1 domains’ three α-helices (αD1a, αD1b, and αD1c) to the concave face of the initial TLR5’s LRR domains (LRR1–5 and LRR8–12 in TLR5-1, and LRR1–5 and LRR8–11 in TLR5-2). This was followed by secondary anchoring of the βD1a sheet and αD1b helix of *ep*flagellin with the LRR domains (LRR15–19 in TLR5-1, and LRR14–19 in TLR5-2) of the secondary TLR heterodimer pair to stabilize the dimerization. TLR5–1 and TLR5–2 consist of 24 LRRs and 23 LRRs, respectively. As a result, each *ep*flagellin interacts with residues of each TLR5 within the dimer. This similar interface is displayed in zebrafish TLR5 homologs binding to *Bacillus subtilis* flagellin, indicating a conserved mechanism of flagellin recognition and TLR5-mediated activation across other teleost species (253).

The minor sequence differences among TLR5 variants result in changes in key residues that affect heterodimer binding, as illustrated by variations in *ep*flagellin’s bonding and anchoring to TLR5. Computationally predicted residue differences in the binding interface between the two homologs included L240 for Q240, R305 for R303, and N88 for N83 between TLR5–1 and TLR5-2, respectively. In the anchoring region, TLR5–1 features an additional interacting residue, Glu451, compared to TLR5-2. Structurally, TLR5–1 includes an additional LRR segment corresponding to LRR12 in the bonding region with αD1b, whereas TLR5–2 features an extra LRR contribution from LRR14 in the anchoring region with βD1a.

### 5.2 Structural basis of CpG DNA recognition by TLR9 and TLR21

TLR21 is absent in mammals but is conserved across teleost fish, amphibians, and birds, serving as a homolog of mammalian TLR9 by recognizing unmethylated CpG motifs (51, 261, 262). In Channel catfish, both TLR9 and TLR21 were involved in immune activation mediated by synthetic cytosine-phosphate-guanosine oligodeoxynucleotides (CpG-ODNs), mimicking microbial CpG DNA, as mentioned (51). Lai et al. (2019) demonstrated that among these, CpG-ODN2007 is a single-stranded DNA (ssDNA) sequence originally characterized in zebrafish, which is a close teleost relative to channel catfish, and induced the strongest activation for both receptors, whereas the other ODNs produced receptor-specific responses *in vivo* (261). Because CpG DNA was not directly extracted or sequenced from catfish tissues, CpG-ODN2007 was used as a standardized synthetic ligand to enable comparative docking and to identify predicted binding interactions that may inform future adjuvant evaluation.

**Figure 4:**
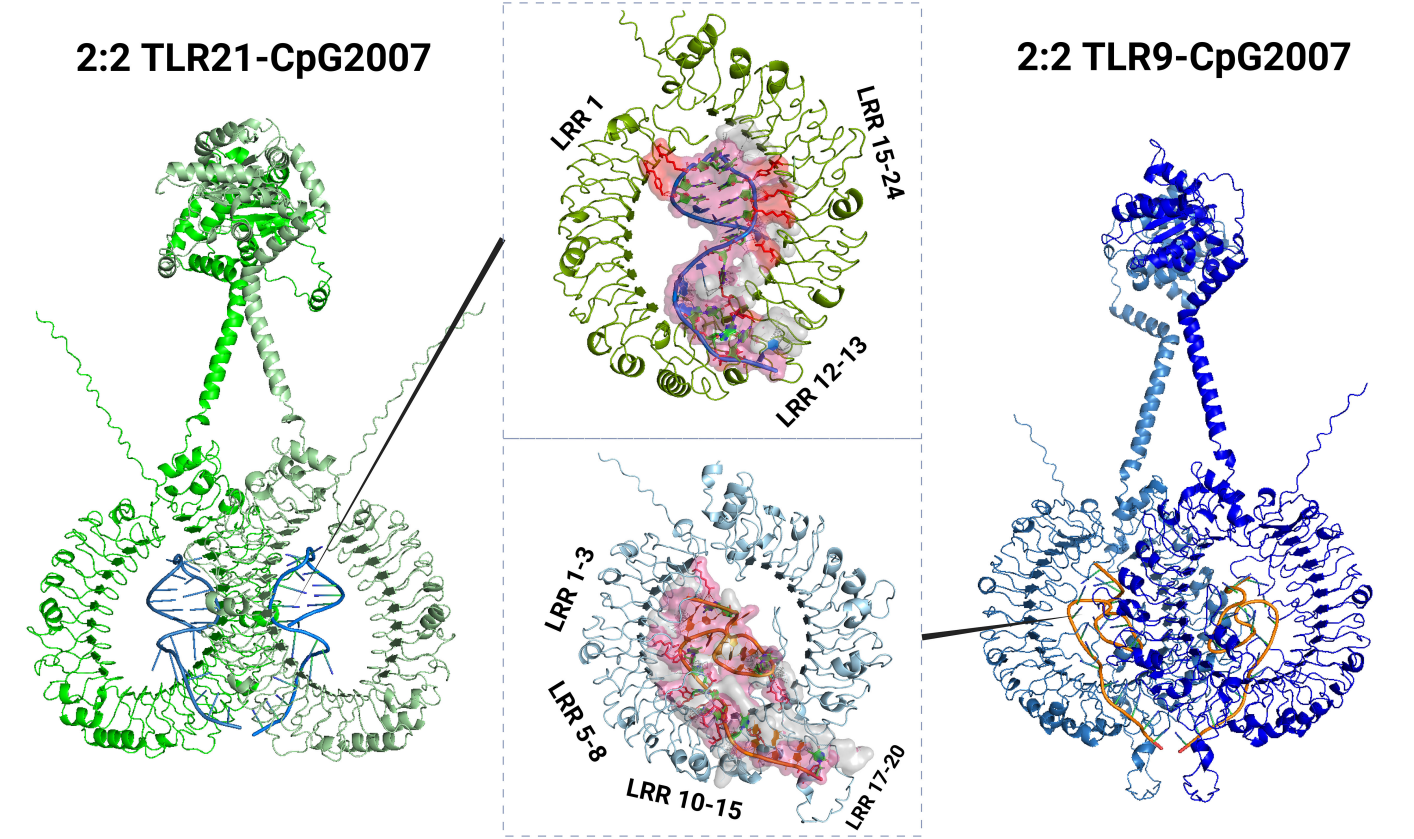
Structural comparison of Channel catfish (*Ictalurus punctatus*) TLR21–CpG2007 and TLR9–CpG2007 2:2 complexes. Modeled ligand-bound homodimers are shown for TLR21 (green) and TLR9 (blue). Each forms a 2:2 complex with CpG-ODN2007 ssDNA (blue/orange helices). Insets show detailed views of ligand recognition interfaces. For each TLR homodimer, CpG-ODN2007 is bound within the concave surface spanning LRRs. Interaction coloring corresponding to hydrogen bond and van der Waals interaction zones follows the scheme described in **Figure 3**. These interactions delineate the ligand-binding interface across the LRR domains, which are labeled according to their position along the ectodomain. The structures underscore conserved 2:2 symmetric architectures but distinct patterns of ligand engagement between TLR21 and TLR9. Protein sequences for TLR9 and TLR21 were retrieved from NCBI under accession numbers AEI59673.1 and AEI59678.1, and CpG-ODN2007 was modeled as a synthetic ssDNA oligonucleotide (5′-TCGTCGTTGTCGTTTTGTCGTT-3′) consistent with its characterization in zebrafish (156). Two separate multimer predictions were run on AlphaFold Server v2.3, each submitting FASTA sequences of two copies of the respective TLR with two copies of CpG-ODN2007 to model each homodimeric complex independently. Both predictions yielded ipTM = 0.76 and pTM = 0.77, with per-residue pLDDT scores predominantly >90 in the LRR binding regions. Structures were imported into PyMOL v3.0 without energy minimization for visualization and figure preparation. Interface contacts were classified using LigPlot+ v2.2 (72). LRR boundaries were identified using LRRsearch v1.0, and all other domains were annotated using SMART v10 (72, 74). The figure was created in BioRender.com (72). All interactions shown are computationally predicted and require experimental validation.

In modeled complexes, TLR21–CpG2007 interactions were displayed spanning the helical concave surface of LRR1 and LRR12–24, while TLR9–CpG2007 binding occurred across LRR1–3, LRR5–8, LRR10–15, and LRR17–20 as shown in **Figure 4**. These patterns suggest distinct ligand-engagement interfaces that warrant experimental confirmation that reflect receptor-specific recognition. This adds to the functional divergence of TLR9 and TLR21 in teleost immunity, which is vital in recognizing variations of pathogenic CpG DNA. Both TLRs were predicted to exhibit positively charged residues interacting with the phosphate backbone, and polar residues contacting unpaired nucleotides of CpG-ODN2007, forming key stabilizing interactions within the binding interface. The mechanism of homodimerization for TLR 9 involves initial monomeric binding to CpG DNA to form a 1:1 complex, followed by a ligand-induced dimerization to yield a stable 2:2 signaling complex, as verified by Reikine et al. (2020), which was used to guide this binding prediction (262). Much like TLR5–1 and TLR5-2, the TM domain of TLR9 and TLR21 is responsible for homodimerization (66).

#### 5.2.1 Signaling pathway of TLR9 and TLR21 activation

Comparative signaling pathways for TLR9 and TLR21 were constructed by compiling experimental data across multiple fish species, incorporating adaptor proteins, transcription factors, and the specific cytokines that have been previously mentioned in the literature. While the TLR9 pathway was based on well-characterized signaling mechanisms, the TLR21 model was inferred from limited teleost data and comparisons with mammalian and avian homologs to propose a likely signaling cascade.

**Figure 5:**
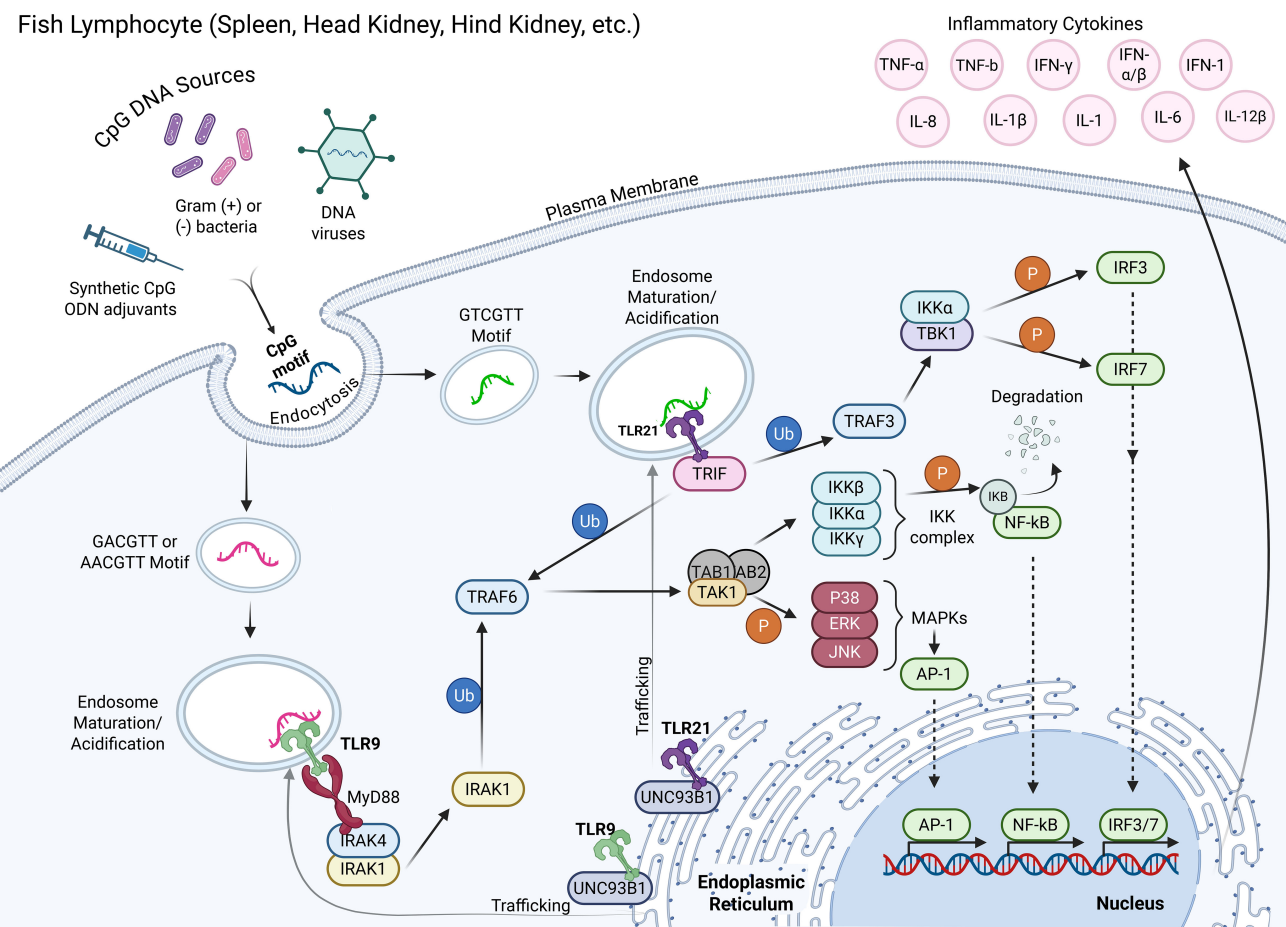
Comparison of fish TLR9 and TLR21 signaling pathways in response to CpG DNA motifs. CpG DNA motifs are DNA sequences from various bacterial, viral, and synthetic sources that contain a cytosine (C) followed by a guanine (G) separated by a phosphate group that stimulates TLRs 9 and 21 in fish. Homodimerization is required for activation of both TLR9 and 21. In TLR9, this binding induces the myeloid differentiation primary response gene 88 (MyD88) -dependent signaling pathway, whereas fish-specific TLR21 has been recently identified to signal through the TIR domain-containing adaptor-inducing interferon-β (TRIF) -dependent pathway. Ubiquitination (Ub) and phosphorylation (P) steps are represented by the blue and orange circles, respectively. Transcription factors Activator Protein 1(AP-1), nuclear factor-kappa B (NF-κB), and Interferon Regulatory Factors 3 and 7 (IRF3 and IRF7) induce the expression of inflammatory cytokine genes to induce an immune response. The Image was created in BioRender.com (72).

CpG oligodeoxynucleotides (ODNs) have been documented to activate immune responses via TLR9 and TLR21 in many teleost species, including grouper, rockbream, flounder, cobia, salmon, yellowtail, croaker, zebrafish, tilapia, turbot, seabass, carp, seabream, and others (51, 261). TLR9 is conserved with its mammalian counterpart, whereas TLR21 is entirely absent in mammals. TLR9 isoforms across fish species vary significantly in LRR count and signaling; for example, in orange-spotted grouper, the TLR9B isoform was discovered to dampen receptor activation by disrupting interleukin-1 receptor-associated kinase (IRAK) 4/6 interactions, whereas mammalian TLR9 predominantly upregulates inflammation (158). As mentioned before, fish TLR21 signals through a MyD88-independent/TRIF-dependent mechanism, contrasting with the MyD88-dependent pathway identified in chickens. Expression patterns between the two receptors also vary significantly in fish, with one experimental study revealing that TLR9 is expressed 10–30 times higher than TLR21 following CpG stimulation in Atlantic Salmon (212). As previously mentioned, there are also disparities in the ligand specificity between the two receptors, with TLR9 binding DNA motifs resembling mouse TLR9 (GACGTT and AACGTT), while TLR21 prefers motifs aligning with human TLR9 (GTCGTT motifs) (154). These findings are included in the proposed comparative signaling model of **Figure 5**.

Our understanding of the comprehensive signaling of TLR9 and TLR21 is as follows: CpG ODNs enter fish lymphocytes by endocytosis, forming the early endosome. Upon maturation, the ectodomain initiates trafficking of TLR9 and TLR21 from the Endoplasmic Reticulum (ER) by chaperone protein UNC93B1 to the mature endosomal membrane (154). CpG ODN binding initiates homodimerization of both TLR9 and TLR21. MyD88-dependent signaling pathways function primarily in TLR9 upon recruitment of the MyD88 adaptor protein, forming the Myddosome as it associates with the IRAKs, ubiquitinating adaptor protein Tumor Necrosis Factor Receptor-Associated Factor 6 (TRAF6). TRAF6 is then responsible for activating Transforming Growth Factor β-activated Kinase 1 (TAK1) and its binding partners, forming a central mediator complex (158). TLR21 signals exclusively through the TIR-domain-containing adapter-inducing interferon-β (TRIF) pathway, where it can similarly activate the TAKs (170). Both receptors can go on to phosphorylate the MAPK pathways and master Inhibitor of kappa-B kinase complex (IKK complex), which stimulates the transduction of transcription factors AP-1 and NF-κB, respectively, to the nucleus, where they allow the transcription of inflammatory cytokines (261). Alternatively, TLR21 phosphorylates TANK-binding kinase 1 (TBK1), where it induces the expression of transcription factors IRF3 and IRF7 that induce the expression of Type-1 interferons (IFN-α/β) that bridge innate and adaptive immunity, as seen in **Figure 5** (170).

## 6 TLR recognition and response to various pathogens

Economic losses experienced globally because of the often-fatal infections are a major argument in favor of advanced vaccine delivery methods that use TLRs and other PRRs, which are essential components of the innate immune system and defense against infections. Due to this expansion of innate immunity and especially TLR ligand recognition in fish, adjuvants and vaccines against these infections should be formulated to target and induce these early immune responses that are often longer and more dramatic than those observed in mammalian species.

This section will cover several of these examples in literature, focusing on the specific TLRs upregulated upon infection, tissue-specificities, and the inflammatory signaling cascades that result. We will cover common gram-positive and gram-negative bacterial infections, as well as different DNA, ssRNA, and dsRNA viruses that induce the viral response expressing a variety of Type-1 interferons and cytokines. Parasitic infections will also be included for their ability to upregulate a broad spectrum of TLRs despite a lack of a “parasitic-specific” TLR. Although a few species have been characterized within the TLR family, fungal infections will be excluded from this section due to their limited characterization and activation of TLRs in fish-specific studies, rather act alongside C-type lectin receptors (25, 263).

### 6.1 Bacteria

There have been extensive studies into fish TLR response to gram-negative bacteria primarily via the LPS TLR4 sensor and the resulting upregulation of pro-inflammatory cytokines. One study examined formalin-killed *A. hydrophila* and its stimulation of TLR1 primarily in the kidney, gallbladder, and gonads of hybrid Yellow Catfish (83). TLR2 was expressed to a similar degree due to its association with TLR1 to form the TLR1/2 heterodimer that forms a complex with MyD88, Fas-Associated Death Domain (FADD), and Caspase-8 to trigger apoptosis and is responsible for expressing inflammatory cytokines TNF-α, IL-1β, IL-6, and IL-12 (83). Upon infection with *Edwardsiella ictaluri*, a commonly encountered bacterium that induces enteric septicemia infections in aquaculture species it has been noted in channel catfish that there is significant upregulation among TLR2, TLR3, TLR5, TLR20a, and TLR21; especially before the 24-hour mark after challenge, suggesting the importance of early intervention (264). Another study, this time in Indian major carp, also observed TLR5M upregulation upon *A. hydrophila*, and *Edwardsiella tarda* infection peaking at ~17-fold and ~5-fold, respectively, alongside a number of adaptor proteins such as MyD88 and TRAF6 (202). The same study found the highest expression of TLR5M to be primarily in the kidney and blood for *A. hydrophila* and liver, kidney, intestine, and blood for *E. tarda*. Infection with gram-negative *Vibrio anguillatum* upregulated the expression of TLR 8 (~75.9 fold in the blood) and TLR 9 genes (~11.8 fold in the brain) in turbot, at varying levels across immune organs. The same study investigated specifically TLR8 upregulation by *V. anguillatum* in comparison to gram-positive *Streptococcus iniae*, where *V. anguillatum* displayed generally higher expression rates primarily in the liver (265).

Gram-positive bacteria lack the LPS-containing outer membrane characteristic of gram-negative bacteria but have a thick, PGN-rich cell wall that induces PGN-specific TLRs like TLR2 as well as coreceptors shared with gram-negative PAMPs. After treatment with *Streptococcus uberis*, TLR2 was upregulated ~4-fold after 24 hours of infection in the liver, as well as adaptor proteins MyD88 (~7-fold in the liver) and TRAF6 (~3.5-fold 24 hours post-infection in the kidneys) in Indian major carp (202). Similar to gram-positive bacteria, TLR5 activity was also observed after *S. uberis* infection, contributing to the expression of the observed pro-inflammatory cytokines like IL-8 and TNF-α. Another study investigating *S. iniae* induced upregulation of TLR 8 and TLR 9 genes in turbot found varying levels of expression primarily in the intestines and skin post infection, with the highest rates at ~47.5 fold in the intestines after 12 hours and the highest expression of TLR9 ~33.9 fold in the intestine after 4 hours (265). These few examples demonstrate how the involvement of a wide range of TLRs in fish is required for effective early immune defenses against commonly encountered bacterial aquatic pathogens that cause significant fatalities in the aquaculture industry.

### 6.2 Viruses

Infections by viral pathogens are a major cause for concern within aquaculture, with few, if any, treatment options once there is an active infection and a limited number of practical, effective vaccines currently available for commercial use (266). Alongside other PRRs like RIG-I-like receptors, NLRs, and cytosolic DNA sensors, viral-sensing TLRs are critical to the early response against these destructive infections (267). TLRs most associated with viral infection may be endogenous, like TLR3, TLR7, and TLR8, or they may be extracellular for recognizing viral surface proteins like TLR4, but other TLRs have also been identified for their role in several aquatic viruses (268).

One commonly encountered example is GCRV, a dsRNA virus that causes severe hemorrhagic disease in several bony fish species, which has been investigated for its upregulation of several TLRs. One study investigated the response to GCRV by barbel chub TLR7 and TLR8, finding that TLR7 was most highly expressed in the brain and spleen and TLR8 similarly expressed in the spleen (148). These TLRs were observed to stimulate expression of type-1 IFNs by the TRIF pathway, inducing the viral response as well as inflammatory cytokines that are positively regulated by the expression of adaptor protein MyD88 (148). GCRV has been a significant concern, especially in the Chinese aquaculture market in recent years, where hemorrhagic disease in the commonly farmed freshwater fish, grass carp, leads to high mortality rates due to insufficient disease management practices and drugs (269). Viral Hemorrhagic Septicemia Virus (VHSV) is a negative-sense ssRNA virus whose infection has caused devastation, especially within the Korean aquafarming industry, where it is highly contagious and causes significant hemorrhaging and major economic losses (270). It’s been studied specifically in olive flounder for its positive and negative regulation of TLR2 and TLR7. Although TLR2 is typically considered for its role in sensing bacterial PGN, it is also capable of binding viral glycoproteins embedded into the outer lipid membrane of enveloped viruses such as VHSV (271). TLR2 and TLR7 are both expressed immediately following infection at relatively high levels, but once the viral load number reached a high threshold, both TLRs were downregulated, implying a suppression of inflammation and overall immune response (272). Inflammatory cytokines and type-1 interferons associated with the viral response were upregulated post infection and include Mx protein, Interferon-stimulated gene 15 (ISG15), IRF7, and IRF3, as well as IL-1β, IL-6, and IL-8 (272). In salmonid fish species, infectious hematopoietic necrosis virus (IHNV), an enveloped, negative-sense single-stranded RNA virus, has been extensively studied due to its significant economic impact on the aquaculture sector, where infection results in high mortality and clinical signs such as exophthalmia, hemorrhaging, and pale gills (273). In rainbow trout, TLR2 was downregulated in head kidney on day 3 post-infection by IHNV, indicating virus-induced immunosuppression by interference with the signal transduction of the type-1 IFN system response, which ultimately restricts the release of pro-inflammatory cytokines. The IHNV M protein was singled out as being the primary inducer of the inflammatory downregulation that disrupts host gene transcription and programmed cell death (274). In both ornamental koi fish and common carp, infection by the highly contagious dsDNA Koi herpesvirus (KHV) leads to gill damage, sunken eyes, and high mortality rates, resulting in high economic losses across both the ornamental and food-production sectors of the aquaculture industry (275). KHV induces not only TLR signaling pathways but also the CLR, NLR, and RLR pathways. With respect to the TLR pathways, upregulation of TLR2 (~10-fold), TLR5 (~6-fold), TLR7 (~6.5-fold), and TLR13 (~6-fold) was observed, leading to a potent immune response in the common carp with the expression of anti-inflammatory Transforming growth factor-beta 1 (TGF-β1) by the MAPK pathways as well as pro-inflammatory IL-10 and TNF-like (276). Although there are such high mortality rates associated with viral infection, there are significantly fewer treatments or vaccines against these pathogens, identifying a need for new strategies targeting members of the innate immune system, like TLRs (277).

### 6.3 Parasites

Infection by protozoan and metazoan ectoparasites and endoparasites remains a major challenge in aquaculture, which is exacerbated by contact with intermediate hosts and the high densities associated with aquafarming productions. Although there is a significant need for new strategies to address these high rates of infection, it is less understood how these pathogens activate and signal within teleost innate immunity as compared to the breadth of information available for viral or bacterial infections with identified TLR-inducing ligands (278). In this section, we focus on protozoan infections due to their frequent and common outbreaks, which cause widespread economic losses (279).

*Ichthyophthirius multifiliis* is a highly contagious protozoan ectoparasite that causes clinical symptoms of small white spots or “ich” accompanied by lethargy and loss of appetite (280). In channel catfish, *I. multifiliis* regulates a variety of TLRs, including TLR1, TLR2, TLR9, TLR19, TLR21, and TLR25. TLR1, TLR2, and TLR25 were all upregulated, peaking at 4.36-fold in the head kidney, 4.85-fold in the skin, and 3.51-fold, respectively. TLR19 and TLR21, however, both displayed a mix of upregulation and downregulation across a variety of tissues at different time points (192). The antigen responsible for binding and the associated TLR activation during *I. multifiliis* infection is a Glycosylphosphatidylinositol (GPI) anchored protein, which is a common PAMP among protozoan pathogens (281).

*Cryptocaryon irritans* is another important ectoparasite that leads to devastating effects on aquaculture populations exhibiting clinical signs like small white cysts on fins and gills, lethargy, and other behavioral abnormalities (282). In orange spotted grouper infected with *C. irritans*, the regulation of specifically TLR2 and its adaptor protein MyD88 was investigated, finding that TLR2 was downregulated in the skin and gills at most of the tested time points, acting as an immune suppressant, whereas it was significantly upregulated in the head kidney and spleen peaking at a ~70-fold and ~10-fold increase at the 6 hour mark. Pro-inflammatory cytokine IL-1β was also expressed highly, reflecting the robust inflammatory response elicited by *C. irritans* (79)*. Trypanosoma carassii* is another protozoan parasite that infects the blood of primarily cyprinid fish species, with limited effective treatments. They also contain characteristic protozoan Glycosylphosphatidylinositol (GPI) protein anchors that act as PAMPs and are recognized by TLR2. In common carp, infection by *T. carassii* leads to inflammatory expression of fish-specific cytokine IL-17A/F2 that recruits and activates neutrophils to regulate the production of antimicrobial peptides, effectively enhancing the fish host’s immune defenses (283).

Parasites are among the most common and impactful pathogens encountered in the aquaculture industry, yet most intervention relies more heavily on management strategies like improving sanitation, chemical treatments, and oral medications rather than vaccines. However, few are available for limited commercial use or are actively under clinical investigation (278).

## 7 Conclusion

TLRs are a class of conserved innate immune receptors that have attracted considerable attention in recent decades due to their complicated and intertwined role in vertebrate immunology. This paper focused on the role of TLRs in pathogen sensing, emphasizing the importance of the LRR, TM, and TIR domains in PAMP interaction, localization, and downstream signaling to initiate an immune response. Nomenclature across the six families in fish is modeled mainly after mammalian nomenclature, which groups receptors based on domain organization and phylogenetic sequence similarities. These ancient receptors were first identified in invertebrates and quickly explored in mammals and other vertebrates, where comparative studies have documented the expansion of TLR proteins, especially in teleost as a result of environmental pressures and a less advanced adaptive immune system relied upon more heavily by higher vertebrates. 2-Dimensional and 3-Dimensional modeling of channel catfish and other vertebrate TLRs illustrates this expansion and the subtle changes in domain architecture that lead to functional differences, especially in the LRR domain. TLR signaling is conserved across vertebrates with signaling occurring through either the TRIF-dependent pathway, initiating the viral response, or the MyD88-dependent pathways to induce the expression of inflammatory cytokines and IFNs, except for TLR4 that signals through either; however, instances where fish TLR signaling diverges from their higher vertebrate homologs were investigated. One such instance is described in the pathway of fish TLR21 that depicts the hypothesized signaling through the TRIF-dependent pathway, despite its avian homolog signaling through the MyD88 pathway. Teleost-specific TLR isoforms and adaptations were detailed in the complete overview of the six TLR families (TLR family 1, TLR family 3, TLR family 4, TLR family 5, TLR family 7, and TLR family 11). The exploration of these families across teleost species through a comparative immunological lens is crucial for describing the origins of these receptors in order to make more informed research decisions by drawing comparisons to their better-understood mammalian models. To detail modes of targeting using known TLR agonists, we constructed hypothetical signaling pathways and molecular docking analyses demonstrating how computational simulations are used to non-experimentally test targets for immune modulation and identify potential adjuvant and vaccine candidates. TLRs are critical for defense against a variety of aquatic bacterial, viral, and parasitic pathogens; beyond simply binding and initiating inflammatory responses, TLRs cooperate with other innate receptors, interact directly and indirectly with members of the adaptive immune system, and control inflammation through negative regulation by novel isoforms.

By integrating this comprehensive TLR characterization alongside computational modeling and docking analysis, this report establishes a foundation for species-specific adjuvant development to accelerate the creation of safe, effective, and cost-efficient vaccines for aquaculture species. In particular, TLR5-targeted flagellin adjuvants and TLR9/TLR21-targeted CpG ODNs represent the most immediately actionable candidates for channel catfish and zebrafish (137, 154, 162, 173). Meanwhile, poly I:C remains a strong broad-spectrum antiviral option across commercially important species including salmon, tilapia, and turbot (198, 200, 216). TLR7/8-targeted imidazoquinolines such as resiquimod show promise for antiviral applications in flounder and pompano, and TLR2-targeted lipopeptides including Pam3CSK4 and LTA offer adjuvant potential for bacterial infections across carp, turbot, and Indian major carp (150, 201, 202, 224, 225, 229, 244).

Advancing our current understanding of Toll-like receptor evolution, structure, and function across teleost species, and referencing their higher vertebrate counterparts to investigate conserved features, is critical for improving the modern methods of disease management in aquaculture. Bacterial, viral, and parasitic infections continue to impose significant economic losses, with an estimated $2.48 billion in annual losses just in the Indian aquaculture sector and rising (284). This also drives increased reliance on antimicrobial use that contributes to the progression of antibiotic resistance that threatens global public health. Despite the availability of several licensed vaccines currently available for aquaculture use, their effectiveness varies between individual species due to the diversity of teleost immune responses, highlighting the need for immunological frameworks like these that account for this variation (285). Aquaculture supplies over half of global fish consumption; thus, it is crucial to global food security that we optimize adjuvants and vaccines for these globally important fish species (286). Ultimately, the structural and signaling framework presented here positions fish TLRs not merely as immunological curiosities, but as actionable targets whose rational exploitation could transform disease management in aquaculture and reduce global dependence on antimicrobials.
